# DNA methylation reader MECP2: cell type- and differentiation stage-specific protein distribution

**DOI:** 10.1186/1756-8935-7-17

**Published:** 2014-08-03

**Authors:** Congdi Song, Yana Feodorova, Jacky Guy, Leo Peichl, Katharina Laurence Jost, Hiroshi Kimura, Maria Cristina Cardoso, Adrian Bird, Heinrich Leonhardt, Boris Joffe, Irina Solovei

**Affiliations:** 1 Department of Biology II, Center for Integrated Protein Science Munich (CIPSM), Ludwig Maximilians University Munich, Grosshadernerstrasse 2, 82152 Planegg-Martinsried, Germany; 2 Wellcome Trust Centre for Cell Biology, University of Edinburgh, EH9 3JR Edinburgh, UK; 3 Max Planck Institute for Brain Research, Max-von-Laue-Str. 4, Frankfurt am Main 60438, Germany; 4 Cell Biology and Epigenetics, Department of Biology, Technische Universität Darmstadt, Schnittspahnstr. 10, Darmstadt 64287, Germany; 5 Graduate School of Frontier Biosciences, Osaka University, 1-3 Yamadaoka, 565-0871 Suita, Osaka, Japan

**Keywords:** MECP2, MBD, Histone modifications, Nuclear architecture, Mouse retina, Retina development, Mouse tissues

## Abstract

**Background:**

Methyl-CpG binding protein 2 (MECP2) is a protein that specifically binds methylated DNA, thus regulating transcription and chromatin organization. Mutations in the gene have been identified as the principal cause of Rett syndrome, a severe neurological disorder. Although the role of MECP2 has been extensively studied in nervous tissues, still very little is known about its function and cell type specific distribution in other tissues.

**Results:**

Using immunostaining on tissue cryosections, we characterized the distribution of MECP2 in 60 cell types of 16 mouse neuronal and non-neuronal tissues. We show that MECP2 is expressed at a very high level in all retinal neurons except rod photoreceptors. The onset of its expression during retina development coincides with massive synapse formation. In contrast to astroglia, retinal microglial cells lack MECP2, similar to microglia in the brain, cerebellum, and spinal cord. MECP2 is also present in almost all non-neural cell types, with the exception of intestinal epithelial cells, erythropoietic cells, and hair matrix keratinocytes. Our study demonstrates the role of MECP2 as a marker of the differentiated state in all studied cells other than oocytes and spermatogenic cells. MECP2-deficient male (*Mecp2*^-*/y*^) mice show no apparent defects in the morphology and development of the retina. The nuclear architecture of retinal neurons is also unaffected as the degree of chromocenter fusion and the distribution of major histone modifications do not differ between *Mecp2*^-*/y*^ and *Mecp2*^*wt*^ mice. Surprisingly, the absence of MECP2 is not compensated by other methyl-CpG binding proteins. On the contrary, their mRNA levels were downregulated in *Mecp2*^-*/y*^ mice.

**Conclusions:**

MECP2 is almost universally expressed in all studied cell types with few exceptions, including microglia. MECP2 deficiency does not change the nuclear architecture and epigenetic landscape of retinal cells despite the missing compensatory expression of other methyl-CpG binding proteins. Furthermore, retinal development and morphology are also preserved in *Mecp2*-null mice. Our study reveals the significance of MECP2 function in cell differentiation and sets the basis for future investigations in this direction.

## Background

Methyl-CpG binding protein 2 (MECP2) was discovered as a protein that selectively binds methylated DNA [1]. Mutations of the *MECP2* gene were later identified as the principal causative factor for Rett syndrome, a severe progressive neurological disorder affecting almost exclusively females [2]. Mild loss of function mutations, duplications, and expression level alterations has also been found in patients with a plethora of neurological and mental phenotypes [3-6]. In mice, deletion of the *Mecp2* gene causes symptoms similar to those of Rett syndrome even when the deletion is restricted to the brain [7-10], while expression of *Mecp2* rescues the Rett phenotype. More effective rescue was achieved through embryonic, compared to early postnatal expression [11-13], whereas targeted expression in postmitotic neurons resulted in asymptomatic mice [12,14]. *Mecp2* mutant mice exhibit abnormalities in the number of synapses [15], the morphology of neuronal processes [16,17], neuronal maturation [16], and the neurophysiological activity of these cells [18,19]. These effects are associated with particular neuron types. For instance, brain stem GABA-ergic neurons are affected, but glycinergic ones are not [20]. Glutamatergic neurons of the brain and their synapses are also affected through the expression level of brain-derived neurotrophic factor (BDNF) [21] which is regulated by MECP2 in a neuronal activity-dependent manner [17,22,23].

The results listed above conform to the conclusion that MECP2 deficiency leads to subtle changes in the expression levels of genes causing diverse and widespread phenotypic changes [24]. There is growing evidence that both *Mecp2-*null astrocytes [25] and microglia [26] affect the dendritic morphology of neurons. Lack of MECP2 causes global histone H3 hyperacetylation in neurons [10,27], which can have different effects on transcription depending on which lysine residues are acetylated. It remains, however, unknown if global histone H3 acetylation levels increase exclusively in neurons or also take place in glia [10,21,27]. Factual data about the phenotypic changes in various tissues of *Mecp2-*null mice are currently insufficient and partially controversial.

In addition to its role in transcriptional regulation, MECP2 appears to be important for maintenance of the general chromatin organization. *Mecp2*-null brain shows a ca. 1.6-fold upregulation in spurious transcription of repetitive DNA, in particular L1 retrotransposons and pericentromeric satellites [27], which have been implicated in maintenance of the nuclear architecture and its formation during cell differentiation [28-30]. In all mouse cells, subcentromeric repetitive blocks, composed of major satellite repeat, form spherical bodies, so called chromocenters that are predominantly located at the nuclear periphery and adjacent to the nucleolus. Remarkably, mouse chromocenters are extremely enriched in MECP2 [1] and the same applies to clusters of human alphoid satellites, also often called chromocenters. There is growing evidence that DNA methylation and MECP2 binding to methylated DNA are pivotal for chromocenter formation and, therefore, the establishment of normal nuclear architecture [31-35]. MECP2 indeed seems to be required for chromocenter fusion during differentiation [8,32,36], although other methyl binding (MBD) proteins can compensate for its absence [31,33,35].

In order to provide better understanding of MECP2 function, we characterized the distribution of the protein in more than 60 cell types of 16 mouse neuronal and non-neuronal tissues by immunostaining. We show that MECP2 is expressed at a very high level in all retinal neurons except rod photoreceptors. The onset of its expression during retina development coincides with massive formation of the neural synapses. We also describe the distribution of MECP2 in other tissues at various stages of development and relate its increased expression to the terminal differentiation of cells. Mice lacking MECP2 show no apparent defects in the morphology and development of the retina, as well as in the nuclear architecture of retinal neurons. Finally, we show that the absence of MECP2 is not compensated by upregulation of other MBD proteins but rather causes their downregulation.

## Results and discussion

We studied mouse tissues because the nuclei of all mouse cells have prominent chromocenters which are convenient for the microscopic approach. The main DNA sequence of chromocenters, major satellite repeat, is present on all autosomes, comprises ca. 10% of whole mouse DNA, contains ca. 50% of the CpG dinucleotides of the whole mouse genome [37], and was shown to bind MECP2 [1]. Therefore, chromocenters can serve as a sensitive indicator of MECP2 expression after immunostaining. To avoid interpretations which might depend only on chromocenters, in all relevant cases, we also studied rat tissues. In contrast to mouse, rat chromosomes do not have large blocks of pericentromeric repeats and therefore do not form noticeable chromocenters in interphase nuclei.

The standard methods of protein-level estimation, such as Western blot analysis routinely used for homogeneous cell cultures, are not really useful for native tissues containing various cell types. Therefore, our method of choice was MECP2 immunostaining on cryosections where we could distinguish different cell types using either histological criteria or cell-specific antibodies (Tables 1 and 2). To avoid false-positive and false-negative results after antibody staining, we used a robust and reliable method developed by us earlier [38,39]. This method allows quick comparison of immunostaining results in the same tissue after various fixation and antigen retrieval times. Polyclonal anti-MeCP2 antibodies, mostly used in the study, do not produce nuclear staining in fibroblasts derived from MECP2-deficient mice (Additional file 1A) and, when applied to Western blot, show expected enrichment of the protein in brain tissue derived from wild-type (WT) mice (Additional file 1B).

**Table 1:** List of antibodies for cell type identification in retina and brain and for recognition of retinal structures

**Antibody abbreviation**	**Antigen transmitter/protein**	**Recognized cells/structures**	**Source, catalogue number**
ChAT	Choline acetyl transferase	Cholinergic amacrine cells	Millipore, AB144P
Calbindin	Calcium-binding protein 28 kD	Horizontal cells	SWANT, #300
GFAP	Glial fibrillary acidic protein	Astroglia	Sigma, G 3893
GABA	Gamma aminobutyric acid	Amacrine, horizontal cells	Sigma, A 2052
GABA-A α1	GABA receptor subunit α1	Bipolar, amacrine, and ganglion cell processes in IPL	Millipore, #06-868
GABA-C	GABA receptor subunit ρ1	Synapses in IPL	R. Enz, MPI for Brain Research, Frankfurt
GAT	GABA transporter	Presynaptic terminals	Abcam, ab426
GAD-65	Glutamic acid decarboxylase (GABA-synthesizing enzyme)	Amacrine, horizontal cells	Chemicon, MAB351R
GAD-67	Glutamic acid decarboxylase (GABA-synthesizing enzyme)	Amacrine, horizontal cells	Abcam, ab26116
GS	Glutamine synthetase	Müller cells (astroglia)	BD Biosciences, #610517
GluR3	Glutamate-gated ion channel (glutamate receptor 3)	Synapses in IPL and OPL	Santa Cruz, sc-7612
GlyT1	Glycine transporter 1	Amacrine cells	Chemicon, AB1770
Iba 1	Ionized calcium binding adaptor molecule 1	Microglia/macrophage	Wako, #019-19741
MAP2	Microtubule-associated protein 2	Neurons	Sigma, M1406
NR1C2	NMDA receptor 1 splice variant C2	IPL and OPL synapses	Chemicon, AB5050P
PKCα	Protein kinase C	Rod bipolar cells	Sigma, P 4334
PKA II β	Human protein kinase A, regulatory subunit II beta	Cone bipolar cells	BD Biosciences, #54720
PSD-95	Postsynaptic density protein 95	Photoreceptors (rods and cones) synapse marker	Dianova, MA1-046
SV2	Membrane of synaptic vesicles	General synapse marker	DSHB, SV2-a1
TH	Tyrosine hydroxylase	Dopaminergic amacrine cells	Immunostar, #22941
VGLUT1	Vesicular glutamate transporter 1	IPL and OPL synapses	Millipore, MAB5502
VGLUT3	Vesicular glutamate transporter 3	Amacrine cells	Millipore, AB5421
Znp-1 (Syt2)	Synaptotagmin II	Cone bipolar cells	Zebrafish International Resource Center, University of Oregon, Eugene, OR, Znp-1

Millipore (Billerica, MA, USA), Swant (Marly, Switzerland), Sigma-Aldrich (St. Louis, MO, USA), Abcam (Cambridge, UK), Chemicon (Billerica, USA), BD Biosciences (Franklin Lakes, NJ, USA), Santa Cruz (Dallas, TX, USA), Wako (Richmond, VA, USA), Dianova (Hamburg, Germany), DSHB (University of Iowa, IA, USA).

**Table 2:** List of antibodies for cell type identification in tissues other than the retina

**Cell type**	**Protein**	**Source, catalogue number**
Smooth muscles	Calponin	Abcam, ab46794
Paneth cells	Lysozyme	Dako, A 0099
Enteroendocrine cells	Secretin	Santa Cruz, sc-26630
Goblet cells	Mucin-2	Santa Cruz, sc-15334
Satellite cells	Pax 7	DSHB

Dako (Troy, MI, USA).

### MECP2 in retinal cell types

**Figure 1:**
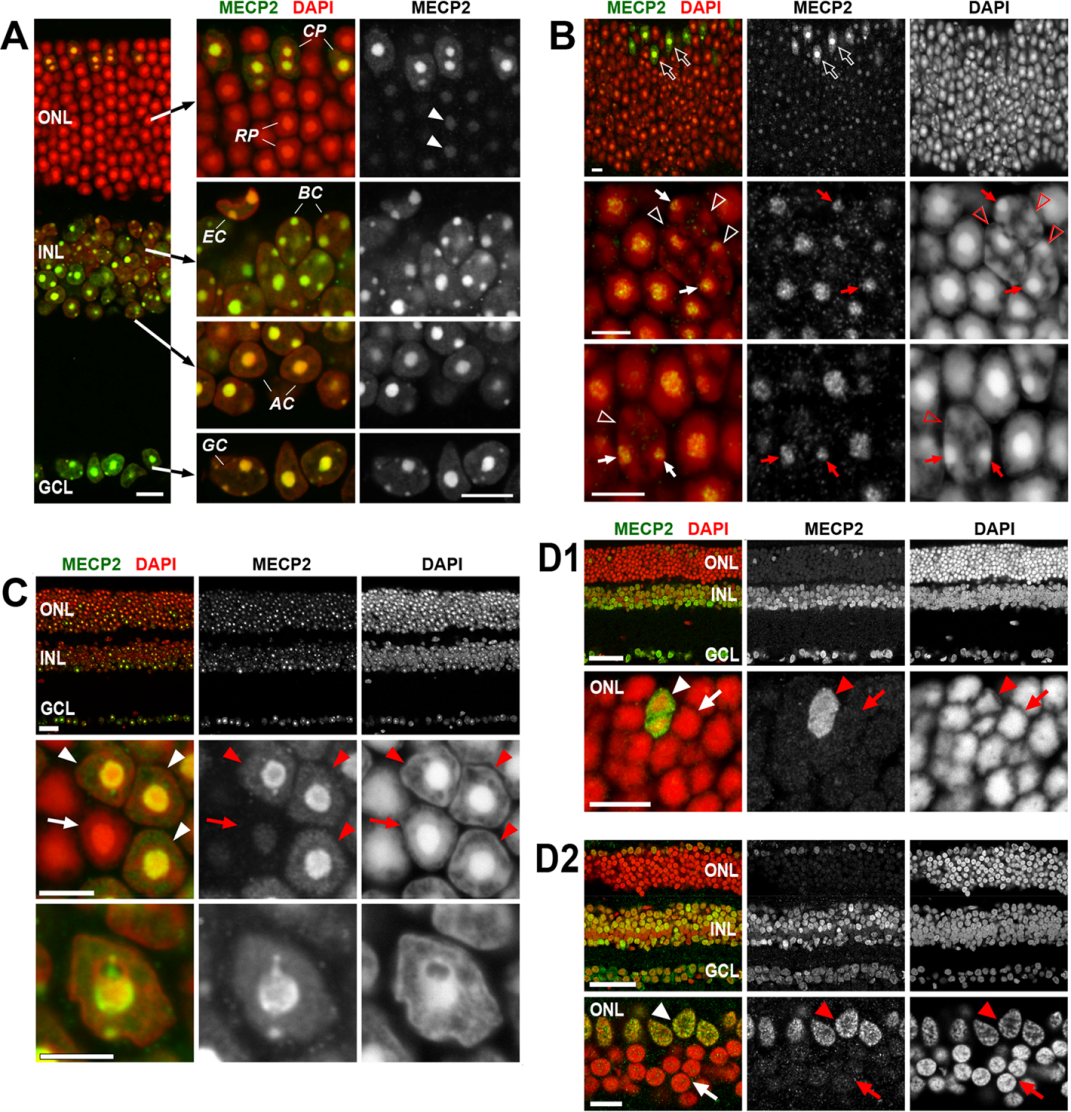
**Distribution of MECP2 in the nuclei of retinal cells. (A)** MECP2 is abundant in all retinal neurons: in the ganglion cell layer (GCL), inner nuclear cell layer (INL), in bipolar (BC) and amacrine (AC) cells. The signal is present throughout the whole nucleoplasm but is especially strong in chromocenters. In the ONL of adult mice, MECP2 produces a strong signal in cone photoreceptors (CP) whereas rod photoreceptors (RP) have very weak staining only noticeable in the chromocenters (*arrowheads*). **(B)** Restoration of conventional nuclear architecture in rod nuclei by *Lbr* expression in Lbr-TER mice does not increase MECP2 expression. In *Lbr*-expressing rods (three such nuclei are marked by *empty arrowheads*), there are multiple chromocenters adjacent to the nuclear periphery. These chromocenters (*arrows*) remain weakly MECP2-positive and with the staining intensity comparable to that of chromocenters in inverted nuclei not expressing *Lbr*. For comparison, bright staining of cone nuclei (*empty arrows*, left and middle upper panels) is shown. Note that all rods with multiple chromocenters adjacent to the nuclear periphery express *Lbr* (Solovei et al. [41]); LBR staining is not shown on this panel. **(C)** In R7E mice, rods de-differentiate, partially restore the conventional architecture of their nuclei, and lose their rod identity. This process is accompanied by increased expression of MECP2 which becomes abundant in chromocenters (three such nuclei are marked by *arrowheads*) and reaches the same level as in neuroretina (upper panel). For comparison, an unaltered rod nucleus is marked (*arrow*). **(D)** Retina of rat **(D1)** and macaque **(D2)**. Similarly to mice, MECP2 produces a bright signal in the GCL, INL, and cones (*arrowheads*) but is weak to undetectable in rod cells (*arrows*). Single confocal sections. Scale bars: (A) 10 μm; (B) 5 μm; (C) overview 25 μm, rods 5 μm; (D) overviews 50 μm, ONLs 10 μm.

The retina is an attractive model to study the role of MECP2 in a nerve center. Most of the retinal cell types can be recognized by their positions and by the shape of their nuclei; only in a few cases, identification requires cell type-specific immunostaining. Most of the mouse retinal cells express MECP2: their nuclei possess a weak or moderate staining of the nucleoplasm and a strong signal in chromocenters. In particular, all neurons in the ganglion cell layer (GCL), inner nuclear layer (INL), and cone photoreceptors in the outer nuclear layer (ONL) have very strong chromocenter staining and a weak nucleoplasm staining (Figure 1A).

In contrast to other retinal cells, rod photoreceptor nuclei of nocturnal mammals possess a dramatically different pattern of chromatin distribution [30]. In these cells, a centrally positioned chromocenter is surrounded by a shell of LINE-rich heterochromatin, whereas euchromatin occupies the nuclear periphery. This nuclear organization is inverted in comparison to all other eukaryotic cells possessing conventional nuclear architecture with heterochromatin abutting the nuclear periphery and euchromatin located in the nuclear interior [28,30]. We have shown that the inverted nuclear architecture in rods has evolved as an adaptation to nocturnal vision: the heterochromatic cores of rod nuclei function as microlenses and reduce light scatter in ONL [30]. Unexpectedly, the nucleoplasm of the inverted rod nuclei is not stained by anti-MECP2 antibodies, and the central chromocenter is only weakly positive (Figure 1A).

In comparison to the multiple chromocenters characteristic of other mouse cell types, the single central chromocenter in mouse rods has a superior chromatin density, which is necessary for rod nuclei to function as microlenses [30]. This high chromatin compaction is obvious from recent electron microscopic studies (e.g., Figure two in [38] and Figure three panel a in [40]) and from the dramatic difference in immunostaining properties between rod chromocenters and chromocenters of other retinal neurons. As described in detail in the recent immunohistochemical studies [38-40], the chromocenter in rods requires much longer antigen retrieval in comparison to the neighboring cones and INL cells. Therefore, to rule out that weak MECP2 staining is caused by inaccessibility of chromocenter chromatin to the antibodies, we made use of transgenic mouse retinas in which rod cells ectopically express lamin B receptor (LBR). Rods expressing transgenic LBR acquire conventional nuclear architecture with euchromatin located to the nuclear interior and heterochromatin, including multiple chromocenters, located at the nuclear periphery. Chromocenters of these transgenic rods have apparently lower chromatin compaction and restore immunostaining ability typical for other retinal cells [41]. However, despite their reduced size and density, chromocenters in LBR-expressing rods remain as weakly MECP2-positive as the chromocenters of wild-type rods (Figure 1B).

The above observations are consistent with results of MECP2 staining in photoreceptors of R7E mice [42]. These transgenic mice specifically express CAG trinucleotide repeat encoding a polyglutamine stretch and represent a mouse model to study spinocerebellar ataxia type 7 (SCA7). In R7E mice, mature rods with inverted nuclei begin to de-differentiate in ca. 1-month-old animals, their nuclei partially restore a conventional nuclear architecture, and photoreceptors lose their rod identity [42]. MECP2 expression in R7E rods gradually increases in parallel to the de-differentiation, and at the age of 20 weeks, the MECP2 level in chromocenters reaches the level observed in the other neurons of the retina (Figure 1C).Furthermore, we also tested for the presence of MECP2 in rods of two other mammalian species: (i) rat, a nocturnal mammal without chromocenters; and (ii) macaque, a diurnal primate with conventional nuclear architecture in rods. In both species, MECP2 was undetectable in rods, in a prominent difference to neuroretinal cells and cone photoreceptors where it produced a clear signal (Figure 1D). Taken together, the above data imply that weak expression of MECP2 is an intrinsic feature of rod photoreceptors.

The low level of MECP2 in rods can be tentatively connected to the relatively high level of linker histone H1c in rod cells described recently for mouse rod photoreceptors [43]. It has been shown that in the MECP2-rich neurons of the brain, approximately half of the linker histone H1 tends to be replaced by MECP2, and that in *Mecp2*-null mice, the H1 level in these neurons doubles [27]. Remarkably, triple KO mice deficient in linker H1c/H1e/H10 histone variants show significant increase of the rod nuclear diameter which was accompanied by decrease of the nuclear volume occupied by heterochromatin. These changes in the nuclear architecture were noticed only in rod nuclei [40]. The other way around, in de-differentiated rods of R7E mice, which demonstrate significantly reduced level of H1c [44,45], the expression of MECP2 increases (Figure 1C).

### Microglial cells have no detectable MECP2

**Figure 2:**
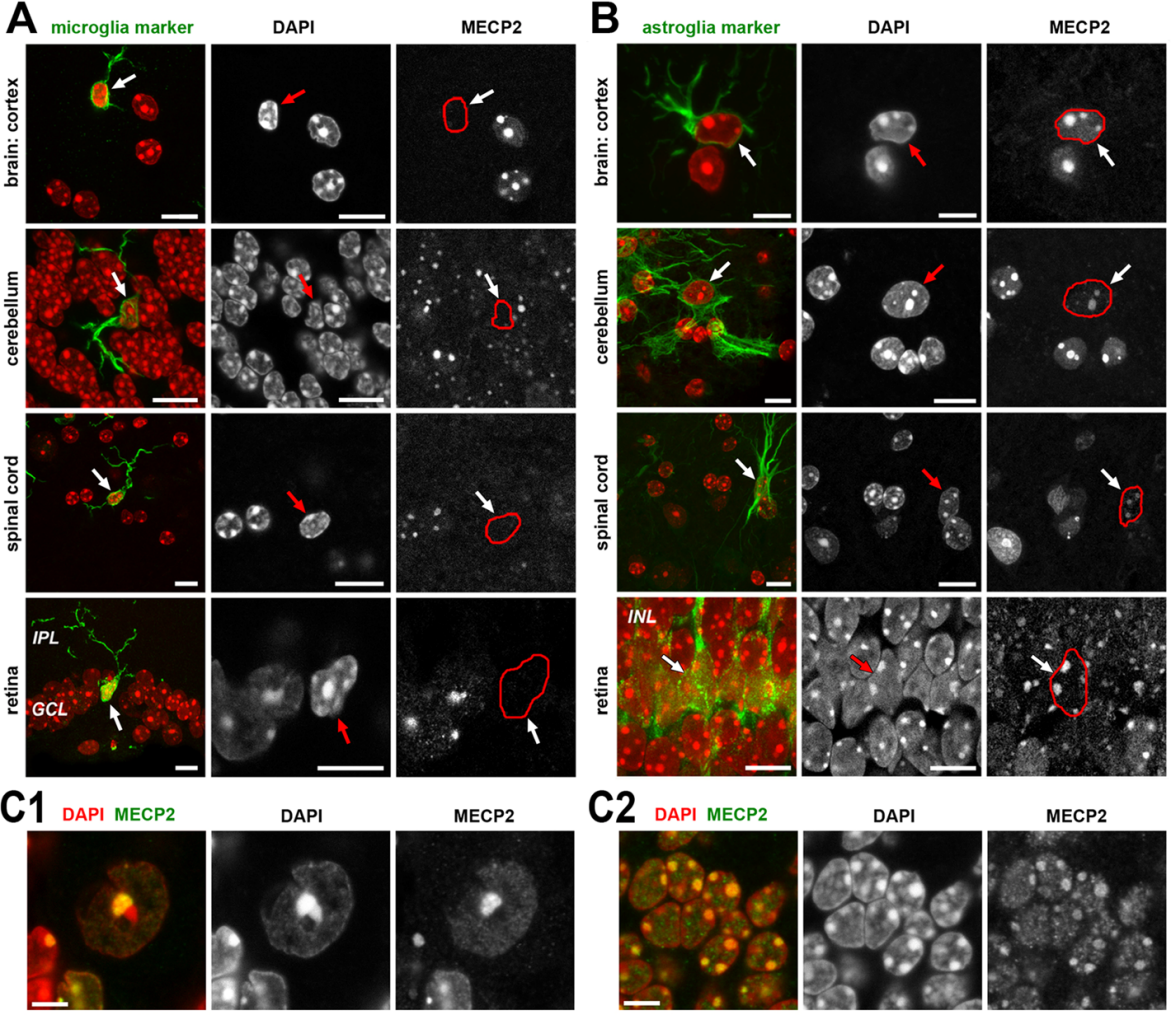
**Microglial cells (A) have no detectable MECP2 compared to astroglia (B) and neurons (C). (A**, **B)** MECP2 detection in brain cortex, cerebellum, spinal cord, and retina combined with microglial **(A)** and astroglial **(B)** cell type-specific staining. Overlays of 4',6-diamidino-2-phenylindole (DAPI) staining (*red*) with markers for microglia (Iba-1) and astroglia (GFAP) are shown in left columns as projections of short stacks. Middle and right columns show single optical sections (zoomed in) for DAPI and MECP2. Non-marked cells in the same images are predominantly neurons and strongly express MECP2. *Red outlines* in the right column images trace the shape of the nuclei of interest. **(C)** Neurons from cerebellum – Purkinje cells **(C1)** and granular cells **(C2)** demonstrate strong MECP2 staining in chromocenters and moderate staining of the nucleoplasm in a single confocal section. Scale bars: (A,B) 10 μm, (C) 5 μm.

Non-neuronal cells of the retina—pigment epithelium, endothelial cells of blood vessels, and Müller cells (radial astroglia)—also expressed MECP2. The only exception was microglia where MECP2 was never detected by immunostaining (Figure 2A). Moreover, microglial cells, identified using anti-lba1 antibodies, were negative for MECP2 staining not only in the retina but also in the brain, cerebellum and spinal cord (Figure 2A). In contrast, in astroglial cells (Figure 2B) and neurons (Figure 2C1,C2), nuclei are strongly positive after MECP2 staining. Absence of MECP2 in microglial cells revealed by immunostaining is especially intriguing in view of recent data on the involvement of microglial cells in the Rett phenotype [46] and questions the role of these cells in neuropathologic consequences of MECP2 deficiency. On the other hand, sensitivity of immunostaining is unquestionably lower than most of biochemical *in vitro* approaches, and therefore, one cannot wholly exclude that microglia cells express MECP2 at a level not detectable microscopically.

### Retinas of *Mecp2*-null mice show no apparent defects

**Figure 3:**
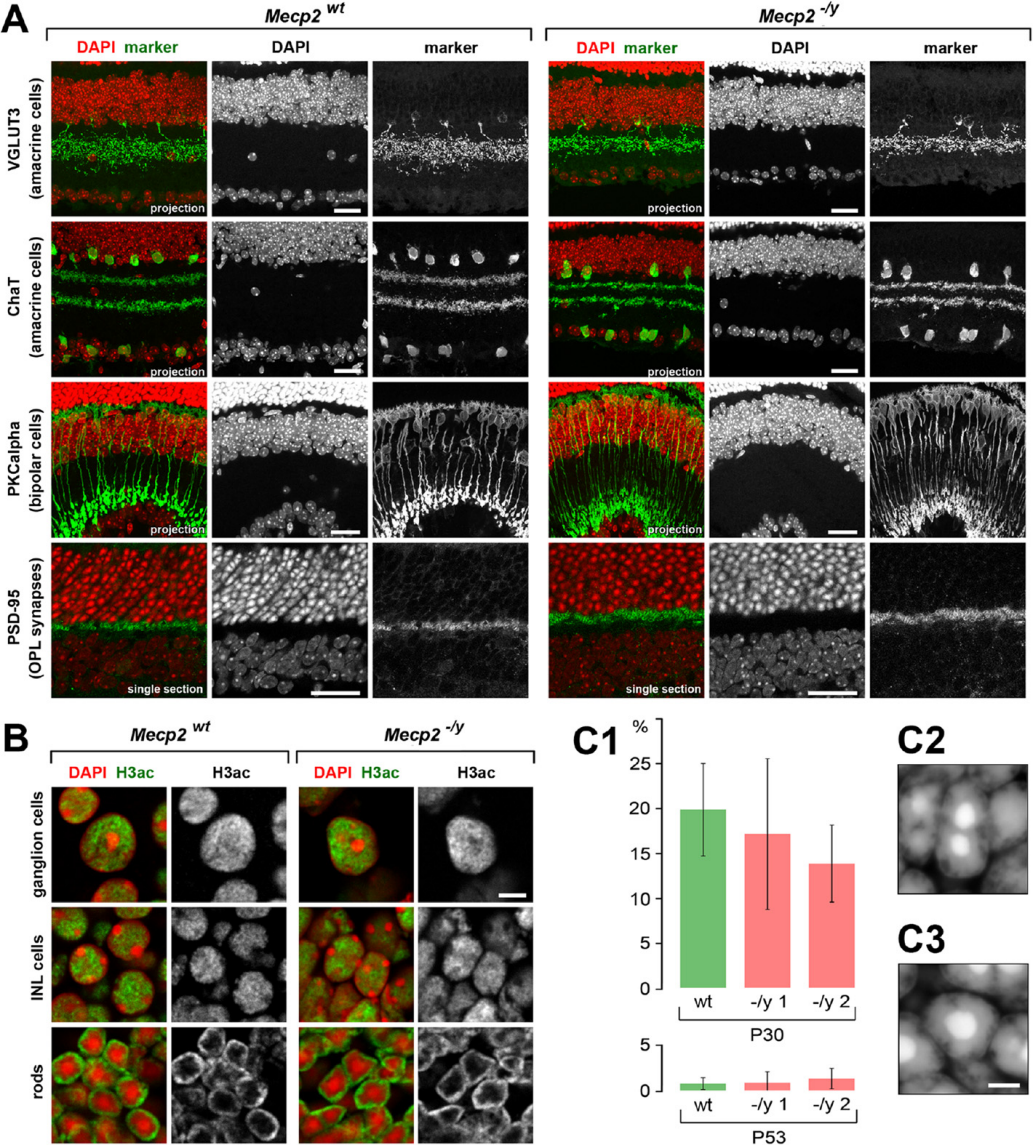
**Retinas of *Mecp2***^**-*/y***^**mice show no apparent defects. (A)** Positioning of amacrine cells, rod bipolar cells, and photoreceptor synapses is similar in retinas of *Mecp2*^-*/y*^ and *Mecp2*^*lox/y*^ littermates. Other 14 markers for retinal cell types, synapses, and neurotransmitters are shown in Additional file 2. **(B)** Similar distribution of a histone modification typical of euchromatin (H3ac) in *Mecp2*^-*/y*^ and *Mecp2*^*wt*^ littermate retinas; nuclei with conventional (ganglion and INL cells) and inverted (rods) architecture are shown. **(C)** The proportions of rod nuclei with two or more chromocenters were scored in retinas of two *Mecp2*^-*/y*^ and one *Mecp2*^*wt*^ littermate at two age points, P30 and P53 **(C1)**. At P53, nearly all nuclei have a single chromocenter. Average proportions of rods with two or less chromocenters were not significantly different between the two genotypes. Errors bars are the 95% confidence intervals. Rod nuclei with two **(C2)** and one **(C3)** chromocenter. Scale bars: (A) 25 μm, (B) 5 μm, (C) 2 μm.

Absence of MECP2 impairs neuronal morphology and strongly affects functions of the brain [9]. The retina, as a compact and very regularly structured part of the CNS, represents an attractive model to study the possible effects of MECP2 on the nervous system development. Earlier, it was shown that in *Mecp2* knockout mice, decline in visual acuity, which was observed in late postnatal development, is caused by general silencing of the cortical circuitry [47]. However, major morphological characteristics of retinas in MECP2-deficient mice have not been yet reported. We dissected retinas of *Mecp2*^-*/y*^ mice at different stages of retina maturation, at postnatal days P1, P7, P13, P30, and P53, and compared their histology to the retinas of wild-type littermates. We found that *Mecp2*^-*/y*^ and WT retinas were not different with respect to the time of layer formation, thickness, and morphology of the layers at all five studied developmental stages (Additional file 2). In addition, we compared *Mecp2*^-*/y*^ and *Mecp2*^*wt*^ retinas with respect to the distribution of various retinal markers. Twelve immunocytochemical markers specific for various amacrine, bipolar, ganglion, and horizontal cells, seven markers for inner plexiform layer (IPL) or/and outer plexiform layer (OPL), and markers for radial glia (Müller cells) and microglia (Table 1) were applied to retinas from adult *Mecp2*^-*/y*^ and WT littermate mice. As shown in Figure 3A and Additional file 3, no noticeable differences in the distribution of certain neurons, synapses, and neurotransmitters were found between the two genotypes.

**Table 3:** List of antibodies for histone modification detection

**Histone/residue**	**Modification**	**Source (catalogue number)**
H3K9	Acetyl	HK (CMA310)
	Me1	HK (CMA316)
	Me2	HK (CMA317)
	Me3	HK (CMA318)
H4K20	Acetyl	HK (CMA420)
	Me1	HK (CMA421)
	Me2	HK (CMA422)
	Me3	HK (CMA423)
H3	Acetyl	Upstate (#06-599)
H4	Acetyl	Upstate (#06-866)

HK produced in the laboratory of Hiroshi Kimura, Osaka University (Osaka, Japan). Upstate (Lake Placid, NY, USA).

### Nuclear architecture of neuronal nuclei in *Mecp2*-null mice is generally preserved

Since MECP2 is a methylation reader and apparently involved in heterochromatin formation [27,36], we checked whether its absence causes changes in the epigenetic landscape of rod and other retinal nuclei. We found that MECP2 deficiency did not have any microscopically visible effect on the presence and distribution of major histone modifications (Table 3). In *Mecp2*^-*/y*^ mice, euchromatin marked by acetylated H3, H4, H3K9ac,me1, and H4K20ac,me1 was present in the nuclear interior of GCL and INL cells and in the outermost peripheral shell of rod nuclei, just as it was observed in WT mice (Figure 3B, Additional file 4). The presence of histone modifications H3K9me2,3 and H4K20me2,3, characteristic of heterochromatin, was restricted to the nuclear periphery and chromocenters of neuroretina cells and was also not different from the wild-type (Additional file 4; see also [38]).

Conversely, we checked whether erasing of the major heterochromatin hallmarks, H3K9me2,3 and H4K20me3, would prevent MECP2 binding. For this purpose, we studied retinas from mice lacking H4K20me3 due to deletion of Suv4-20 h2 and mice lacking both H4K20me3 and H3K9me3 due to deletion of Suv4-20 and Suv3-9 h1,2 methyltransferases. In mice of both genotypes, rod nuclei had the same morphology as the rod nuclei in the wild-type littermate controls [38]. We found that the pattern of MECP2 staining was not different between the retinal cells in the wild-type and knockout mice, suggesting that MECP2 binding to chromatin was not affected. Indeed, MECP2 was strongly expressed in neuroretina and cones, where it localizes mostly in chromocenters, and was almost undetectable in rods (Additional file 5). Recently, it was shown that deletion of Suv4-20 h2 influences chromatin organization in cultured cells, in particular, it increases the number of chromocenters in cultured fibroblasts derived from a *Suv3-9/Suv4-20 h* double knockout mouse [48]. In contrast, double knockout of *Suv3-9* and *Suv4-20* affects neither rod nuclear morphology [38] nor MECP2 binding patterns (this study), suggesting that cells in a tissue context might have more redundancy in epigenetic mechanisms than cultured cells.

Although even a complete loss of MECP2 does not prevent chromocenter formation in mouse cells [8], observations on astroglial cells and neurons differentiated from embryonic stem cells *in vitro* showed that the number of chromocenters was significantly higher in MECP2-null cells compared to wild-type cells [36]. The other way around, ectopic expression of MECP2 induces clustering and fusion of chromocenters, a process which takes place during myotube differentiation [31]. These findings prompted us to assess rod chromocenter numbers in adult mice of both genotypes. Chromocenter fusion in nuclei of mouse rods is a slow process. A significant proportion of rods at ca. 1 month still have two or more chromocenters; their fusion in all rods is completed only at 2–2.5 months of age ( [30,41]; c.f. Figure 3C2,C3). We scored cells with one and two chromocenters in rod nuclei of *Mecp2*^-*/y*^ mice and their wild-type littermates at P30 and P53 (see the ‘Methods’ section for detailed description). The number of rods with two or more chromocenters in *Mecp2*^-*/y*^ mice of these ages was 15.5% at P30 and 1.2% at P53, which was not different from the wild-type (Figure 3C1).

In full agreement with our observations on rod cells, data obtained from cortical neurons in tissue sections and primary neuronal cultures indicate that chromocenter number is comparable between neurons from *Mecp2*^-*/y*^ and *Mecp2*^+*/y*^ mice [35]. Apparently, the difference in results obtained on cells in native tissues of *Mecp2*^-*/y*^ and *Mecp2*^+*/y*^ mice and on cultured cells derived from these mice [36] is analogous to the observations on *Suv3-9/Suv4-20 h* double knockout cells and might be tentatively explained by compensatory mechanisms operating *in vivo* but not *in vitro*.

### Almost all cell types in adult mammalian tissues express MECP2

**Figure 4:**
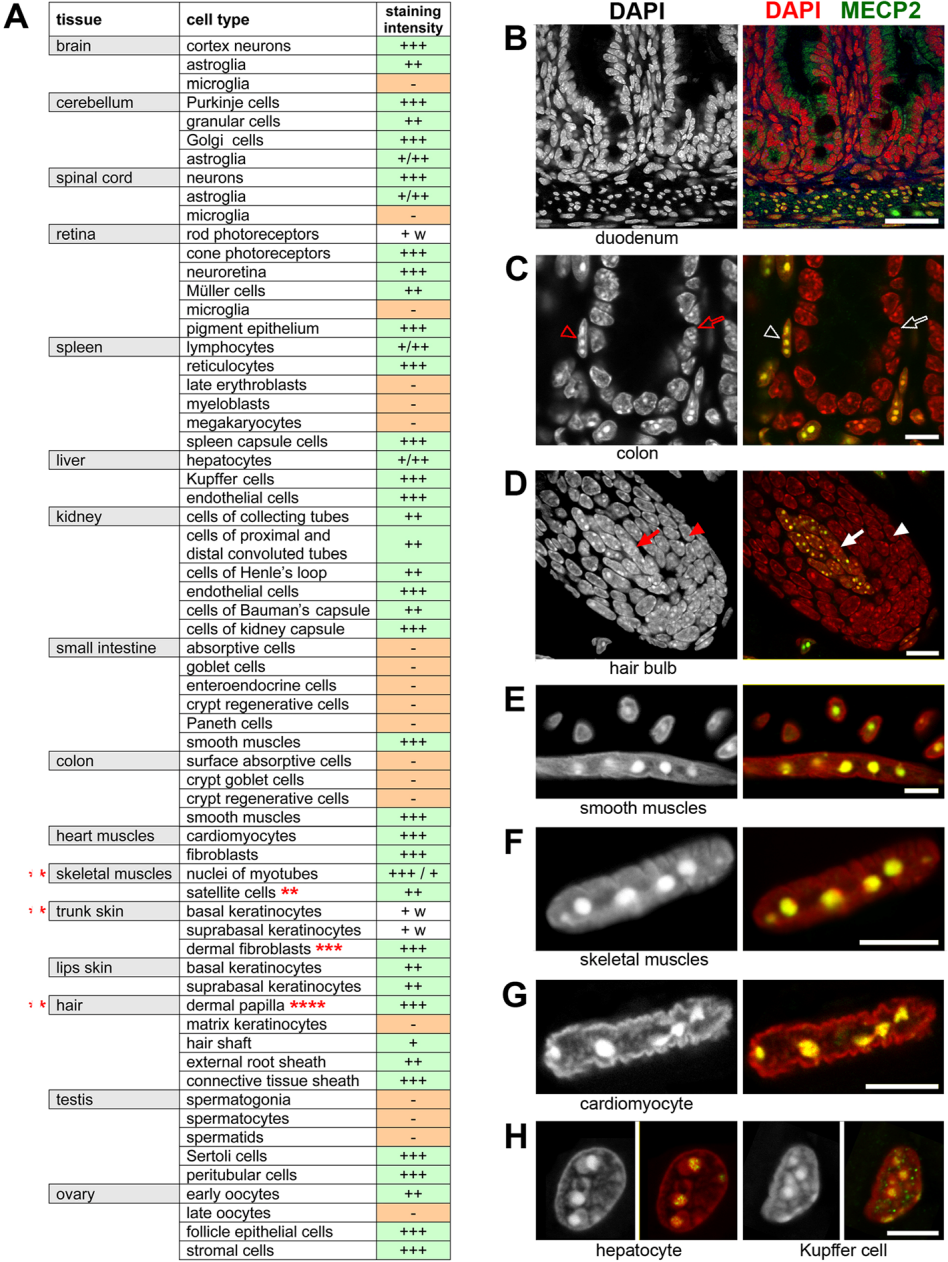
**Presence of MECP2 in different cell types of adult mouse tissues. (A)** List of the studied tissues and cell types; the strength of MECP2 signal is shown by the number of plus signs (1 to 3). *Tissues studied at six developmental age points (P0, P2, P5, P9, and P14). **Satellite cells were negative at P0–P14. ***Dermal fibroblasts were negative at P0–P5. ****Fibroblasts of dermal papilla were negative at P0 and weakly positive at P2; see also Figure 5D. Examples of mouse tissues after MECP2 staining: intestine **(B,C)**, hair **(D)**, muscles **(E,F,G)**, and liver **(H)**. In **(C),***empty arrows* point at MECP2-negative gastroepithelial cells in colon crypt; *empty arrowheads* point at positive smooth muscle nucleus beneath the gastrodermis. In **(D)**, *solid arrows* mark fibroblasts of the dermal papilla; *solid arrowheads* mark matrix keratinocytes of the hair bulb. For comparison of MECP2 staining in mouse and rat tissues, see Additional file 4. Single confocal sections. Scale bars: (B) 50 μm, (C, D) 10 μm, (E, F, G, H) 5 μm.

**Figure 5:**
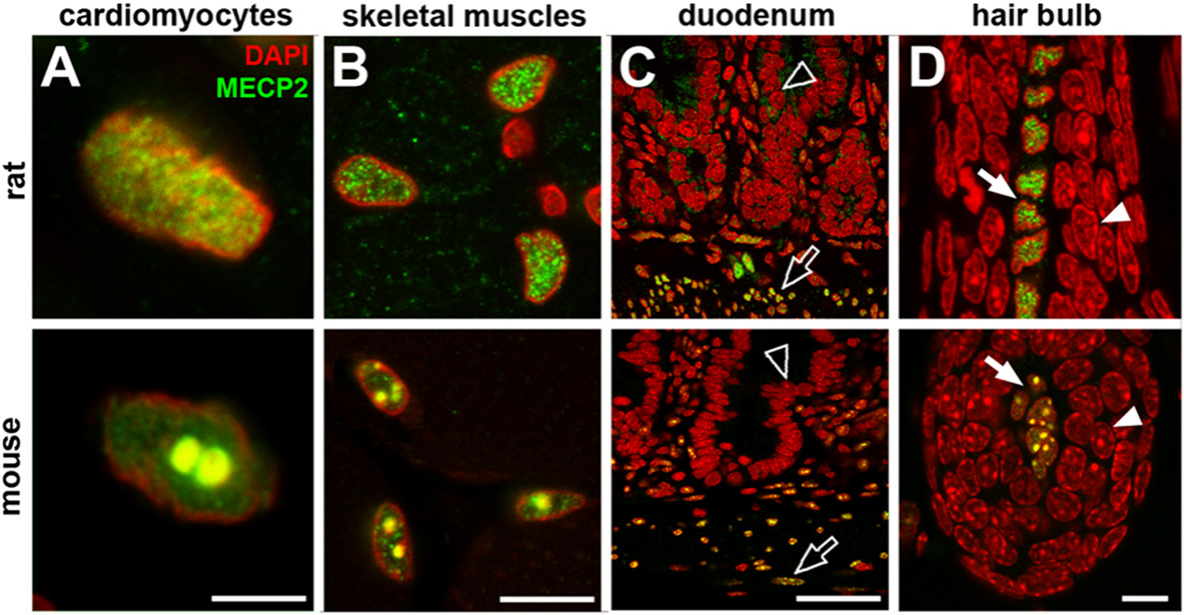
**Comparison of MECP2 staining in selected mouse and rat tissues.** Nuclei of striated muscle cells (**A**, cardiomyocytes; **B**, skeletal myotubes), smooth muscles (**C**, ***empty arrows*** in duodenum), and fibroblasts of dermal papilla (**D**, ***solid arrows***) have strong MECP2 signal in both species. Similarly, gastrodermal epithelial cells (*empty arrowheads*) and matrix keratinocytes (*solid arrowheads*) lack MECP2 in both species. Single confocal sections. Scale bars: (A) 5 μm, (B, D) 10 μm, (C) 25 μm.

The absence of MECP2 in microglia and its low level in rods raised the question of how common MECP2 is in various cell types. Data on MECP2 expression in different tissues are limited, and most reports are based on a bulk analysis of protein or RNA extracted from a whole tissue (e.g., [49,50]). Analyses of specific cell types are only occasional and predominantly concern neuronal tissues [49-51]. Therefore, we studied MECP2 distribution across a number of mouse cell types. Cell identification was based either on histological criteria or, when needed, on cell type-specific immunostaining (for the list of antibodies used, see Table 2). Altogether, about 60 cell types were studied from 12 non-neuronal adult mouse tissues. In addition, epidermis and skeletal muscles were studied at five age points (P0, P2, P5, P9, and P14). The results of immunostaining are summarized in Figure 4A, and telling examples are shown in Figure 4B,C,D,E,F,G,H. We found that the majority of cell types express MECP2; those that do not are rather a minority. MECP2 is lacking in epithelial cells of the intestine and colon. In epidermis, the expression of MECP2 varies: it is absent or present at a hardly detectable level in keratinocytes of the trunk skin but is more abundant in lip epidermis cells, both basal and suprabasal. In the hair, proliferating matrix keratinocytes of the hair bulb lack MECP2 in clear difference to differentiated keratinocytes of hair shaft and hair root sheath where MECP2 produces a clear signal. MECP2 is also not expressed in the erythropoietic lineage, in contrast to other cells of the myeloid lineage and lymphocytes. A noteworthy exception are resident macrophages. As mentioned before, microglial cells in all studied nervous tissues do not express MECP2 at a detectable level (Figures 2A and 4A), whereas resident macrophages from other tissues, in particular, hepatic Kupffer cells, do express it (Figure 4A,H).As MECP2 is primarily visible in the chromocenters of mouse cells, we studied MECP2 distribution in tissues of a species, which does not possess chromocenters in interphase nuclei. Rat chromosomes, in difference to mouse chromosomes, lack large blocks of pericentromeric satellite sequences, and consequently, rat nuclei have no clear chromocenters. Rat small intestine, skin with hairs, and skeletal and heart muscles were studied. Staining of these tissues confirmed that the gastrodermal epithelial and hair matrix cells in rat, similarly to mouse, lack MECP2, whereas the nuclei of muscle cells (smooth, skeletal, and heart muscles) had a strong punctate MECP2 signal in the nucleoplasm (Figure 5). Our data support the notion that in addition to the functions in the nervous system that are associated with a major pathologic phenotype, MECP2 plays some important roles in almost all non-nervous tissues.

Involvement of MECP2 in chromatin regulation and maintenance of global nuclear architecture is well documented [27,52,53]. In particular, it is known that MECP2 plays a role in the regulation of transcription, being mostly a transcriptional repressor [54-56] and also an activator [54]. In the light of these findings, the fact that some cell types across different species are lacking MECP2 is intriguing and requires further analysis.

### Expression of MECP2 increases during tissue development and terminal cell differentiation

**Figure 6:**
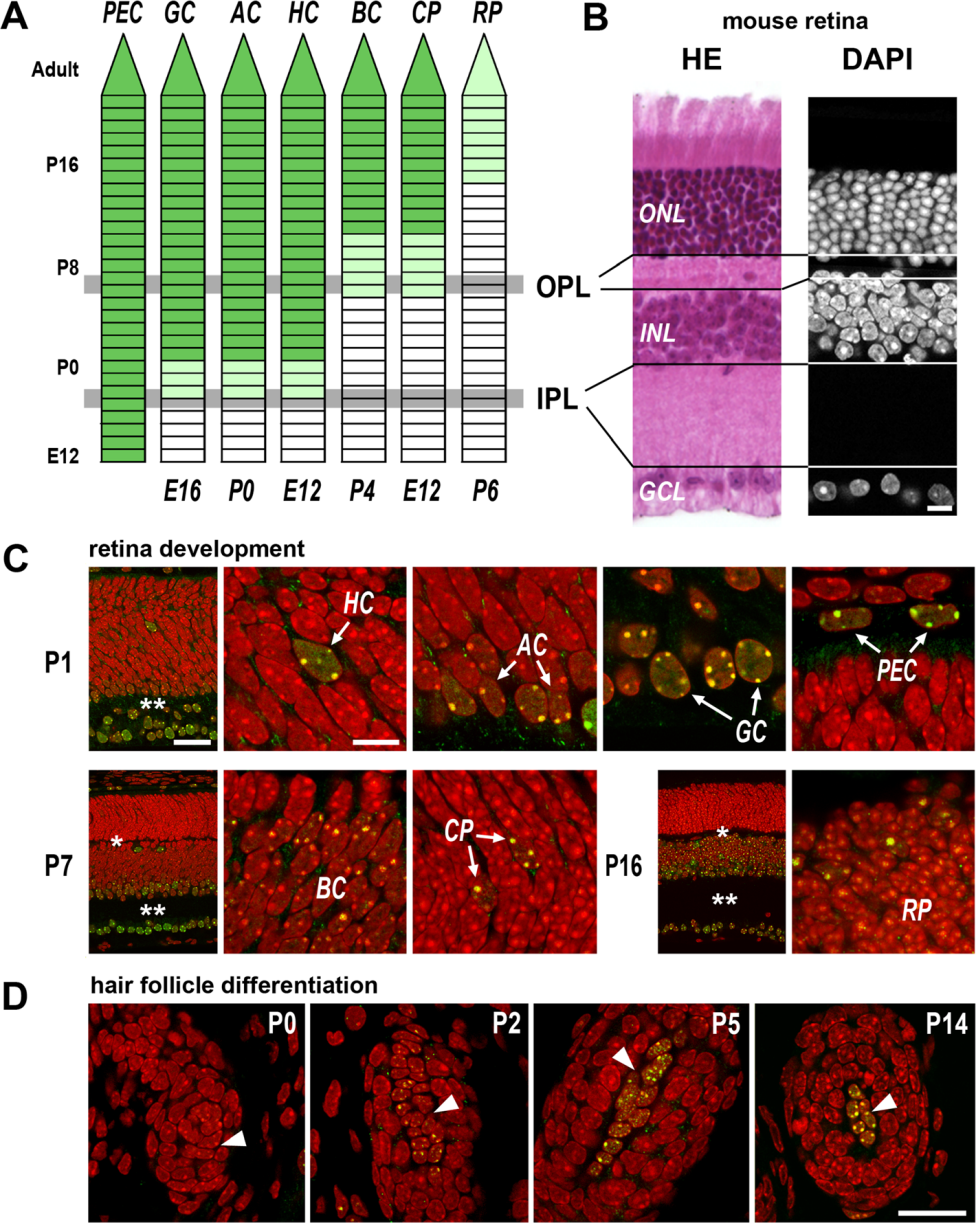
**Expression of MECP2 during development and terminal cell differentiation. (A)** The onset of MECP2 expression (*green*) in different cell types of mouse retina. Time lines are shown for pigment epithelial cells (PEC), ganglion cells (GC), amacrine cells (AC), horizontal cells (HC), bipolar cells (BC), cone photoreceptor (CP), and rod photoreceptor (RP). On the left, postnatal age points are shown; numbers below the time lines show the cell birthdays (the day of the last cell division; [60]). *Grey horizontal lines* mark age points when the outer and inner plexiform layers (OPL and IPL, respectively) become detectable (see also [57-59]). Light *green marks* a low MECP2 level. The onset of MECP2 expression in neurons coincides with massive formation of synapses and, consequently, IPL and OPL plexi. **(B)** Arrangement of the nuclear and plexiform layers in mouse retina revealed in a paraffin section after hemalaun-eosin staining and in a cryosection after nuclear counterstain with DAPI. The perikarya of GCs are located in the GCL; those of BCs, ACs, and HCs are in the INL; and those of the photoreceptors are in the ONL. **(C)** Examples of retinal cells (marked by *arrows*) with initiated MECP2 expression at three age stages. *Single* and *double asterisks* mark OPL and IPL, respectively; the abbreviations are the same as in (A). For comparison with adult mouse retina, see Figure 1A. **(D)** In the fibroblasts of the dermal papilla (*arrowheads*) of the hair follicle, MECP2 expression is initiated postnatally and becomes detectable at P2; later, the MECP2 expression in these cells remains stably high (see also Figure 4A,D). (C, D) Single confocal sections. Scale bars: (B) 10 μm; (C) overviews 50 μm, close-ups 10 μm; (D) 25 μm.

There is a clear difference between MECP2 expression levels in tissues of different developmental stages. A telling example are fibroblasts of the dermal papilla in the hair bulb. These cells lack MECP2 at the late embryonic stages and in the first 2 days of postnatal development; the expression starts at P2 and continues afterwards (Figure 6D).

The expression of MECP2 in the retina starts at different times depending on the cell type. Remarkably, the onset of expression coincides with massive formation of synapses and, as a consequence, the formation of the IPL and OPL [57-59] (Figure 6A,B). In particular, MECP2 appears in the ganglion and amacrine cells at E17, when a clear gap appears between the GCL and INL + ONL anlage, marking the emerging IPL. Similarly, the MECP2 expression in the bipolar cells starts at P6 together with the formation of the gap between the INL and ONL, which develops into the OPL later. In rods, weak MECP2 expression starts after 2 weeks of postnatal development and remains weak thereafter (Figure 6A,C). Noteworthy, the onset of MECP2 expression roughly correlates with cell birthdays (the day of the last cell division; [60]) of the retinal neuronal cell types (*R*_Spearman_ = 0.62) and persists afterwards.

**Figure 7:**
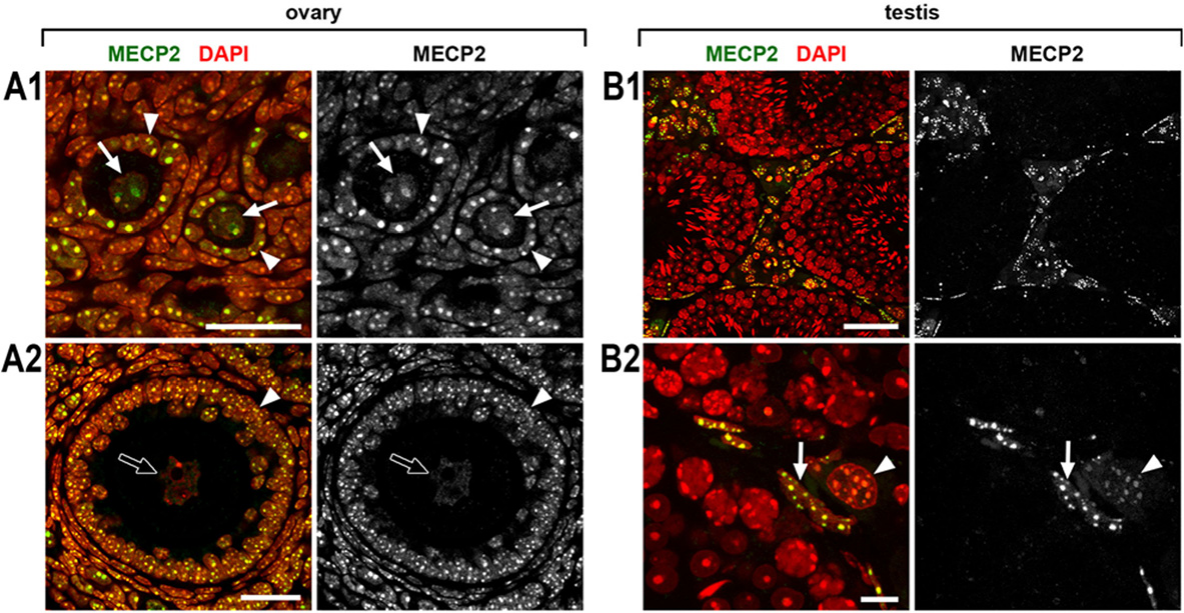
**Expression of MECP2 in the ovary (A) and testis (B).** Only young oocytes (**A1**, ***arrows***) express MECP2; the more mature oocytes **(A2)** do not express MECP2 (**A2**, ***empty arrow***). Neighboring follicular cells (*arrowheads*) strongly express MECP2. In testis, only Sertoli cells (**B2**, ***arrowhead***) and fibroblasts (**B2**, ***arrows***) express MECP2; spermatocytes at all stages of maturation and sperm cells are MECP2-negative. Single confocal sections. Scale bars: (A1, A2) 25 μm, (B1) 50 μm, (B2) 10 μm.

Initiation of MECP2 expression at late differentiation stages proved to be a general rule: undifferentiated or weakly differentiated cells (progenitors) do not express MECP2 or show a low expression level compared to the respective fully differentiated cells. In particular, matrix keratinocytes of the hair bulb do not express MECP2, the more differentiated keratinocytes of the hair shaft show a weak expression, and a stronger expression is observed in the keratinocytes at the root hair shaft. MECP2 is weak in satellite cells but abundant in the myotube nuclei (Figure 4A,F). The reverse situation occurs only in the gonads. In the ovaries, the follicle epithelium and the youngest oocytes express MECP2, whereas mature oocytes do not (Figure 7A). Sertoli cells and fibroblasts are MECP2 positive, whereas spermatogenic cells do not express MECP2 at any stage (Figure 7B). The absence of MECP2 immunostaining in mature gametes conforms to the known fact that zygotes, stem cells, and cells of young embryos [61-63] lack MECP2. In summary, our results indicate that MECP2 is a marker of the differentiated state.

### Absence of MECP2 is not compensated by altered expression of other MBD proteins in cultured cells and native tissues

**Figure 8:**
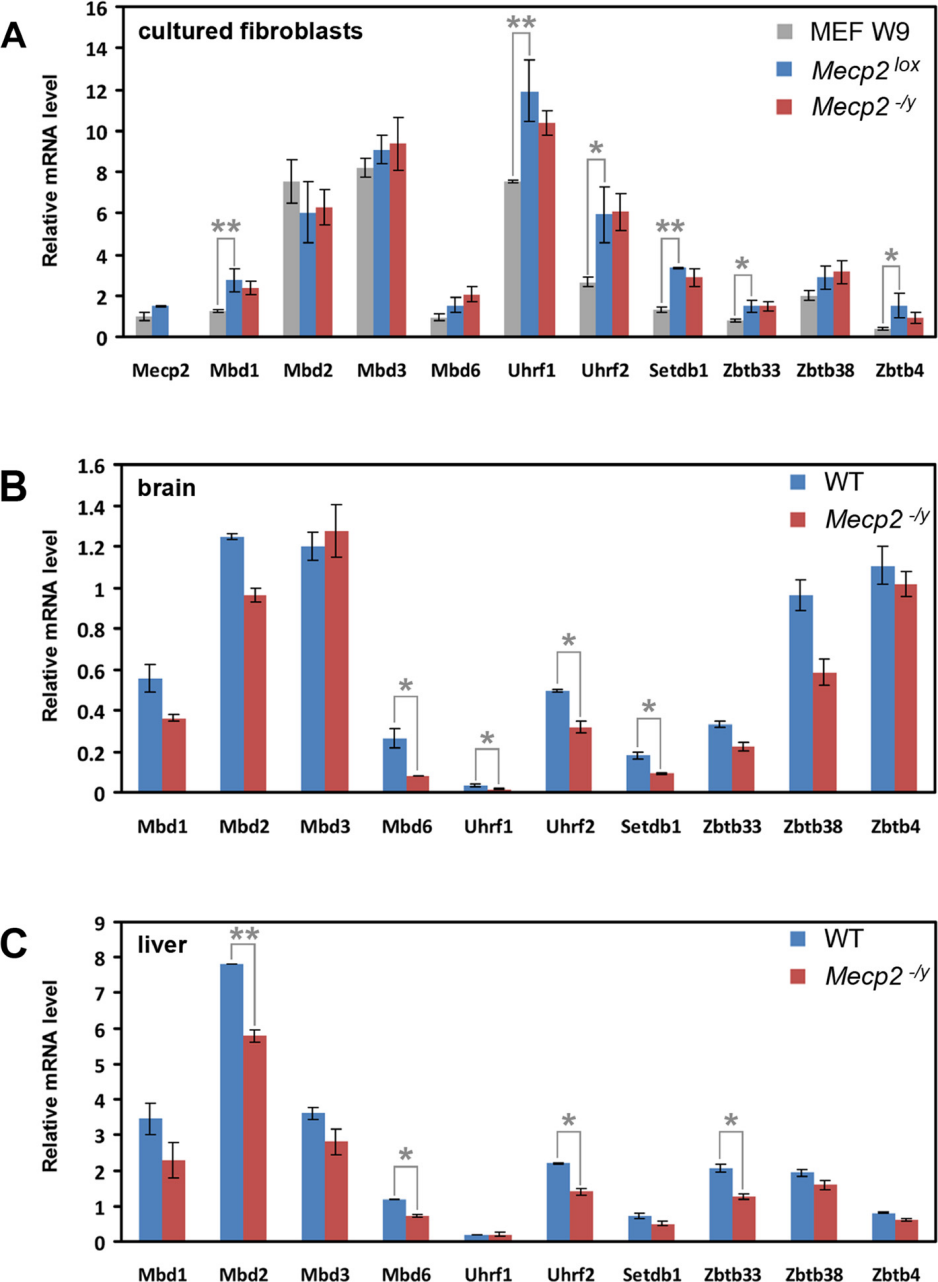
**Analysis of expression of MBD proteins in cultured fibroblasts and tissues from *Mecp2***^**-*/y***^**and wild-type mice. (A)** Relative transcription level of MBD proteins in wild-type embryonic fibroblasts (MEF W9) and adult fibroblasts established from *Mecp2*^-*/y*^ and littermate *Mecp2*^*lox/y*^ mice. Values are normalized to the *Mecp2* transcript in the embryonic fibroblasts. Note that the mRNA levels in the embryonic and adult fibroblasts differ, whereas no difference in transcription was detected between *Mecp2*^-*/y*^ and *Mecp2*^*lox/y*^ genotypes. Relative transcription level of MBD proteins in the brain **(B)** and liver **(C)** from *Mecp2*^-*/y*^ and littermate *Mecp2*^*wt*^ mice. Values are normalized to the *Mecp2* transcript in the respective *Mecp2*^*wt*^ tissue. Note that there is no upregulation of MBD protein genes upon deletion of *Mecp2*. Results of real-time PCR analysis of two (for tissues) and three (for cells) biological replicates are given as mean ± S.E.M. Statistical difference between values was estimated by *t* test; statistically significant differences in transcription levels are marked by *asterisks* (*<0.05; **<0.01).

Considering the specific binding of MECP2 to methylated DNA, we questioned whether other proteins are able to replace MECP2 on 5-methylcytosine (5mC) in case of its absence. Though this has not been systematically investigated, the question has been addressed genetically by Caballero and co-authors [64]. The authors showed that simultaneous deficiency of three methyl-CpG binding proteins MECP2, MBD2, and KAISO in mice is compatible with normal embryogenesis and provided evidence for redundancy of function between these proteins in postnatal mice. Since antibodies to other methyl-CpG binding proteins reliably working on cryosections are lacking, we quantitatively studied the expression level of all known 5mC-binding proteins in *Mecp2*^-*/y*^ cultured cells and tissues by reverse transcription quantitative polymerase chain reaction (RT-qPCR). We focused on an expression analysis of the following methyl binding proteins: four MBD proteins, MBD1, MBD2, MBD3, and MBD6 (MBD4 and MBD5 were omitted due to the nearly undetectable expression level); UHRF1 and UHRF2; SETDB1; and three methyl-CpG binding zinc finger proteins, namely, ZBTB33, ZBTB38, and ZBTB4. First, we analyzed the expression of all the above genes in adult *Mecp2*^-*/y*^, adult *Mecp2*^*lox/y*^, and embryonic wild-type fibroblasts. The analyzed genes were transcribed at different levels in embryonic and adult fibroblasts. In particular, we noted a statistically significant decrease in the expression of *Mbd1* and *Mbd6*, *Uhrf1* and *Uhrf2*, *Zbtb33* and *Zbtb4*, and *Setdb1* in the embryonic fibroblasts compared to the adult cultured fibroblasts. However, we found no apparent difference in gene expression between the adult *Mecp2*^*lox/y*^ and *Mecp2*^-*/y*^ fibroblasts (Figure 8A). Similarly, comparison of gene expression in the skeletal muscle, heart, and small intestine did not reveal any differences between tissues from *Mecp2*^-*/y*^ and *Mecp2*^*wt*^ mice (Additional file 6). Unexpectedly, in the *Mecp2*^-*/y*^ brain and liver, the expression of these proteins (e.g., MBD2) was even significantly decreased (Figure 8B,C). Thus, we demonstrated that absence of MECP2 is not compensated by any other known 5mC binding protein at least at the mRNA level.

## Conclusions

Based on the above discussion, the following conclusions were made:

•All retinal neurons, except rods, express MECP2 at a high level and the onset of its expression coincides with neuron differentiation, in particular, with massive formation of neural synapses in the inner and outer plexiform layers.

•Low expression of MECP2 in rod photoreceptors was found in both the inverted rod nuclei of nocturnal mammals and the conventional rod nuclei of diurnal mammals. We relate this fact to an unusually high level of histone H1c in these cells in comparison to other retinal neurons [43].

•MECP2 is not detectable by immunostaining in the retinal microglial cells, nor in the microglia of the cortex, cerebellum, and spinal cord. In contrast to microglia, the astroglial cells in all neuronal tissues express MECP2 at a level comparable to that in neurons.

•The retina of *Mecp2*-null mice shows no apparent defects in the timing and morphology of the nuclear and plexiform layer formation. No noticeable difference in the distribution of certain neuron types, synapses, and neurotransmitters was found between *Mecp2*-null and wild-type retinas.

•The nuclear architecture of the neuroretinal cells and rod photoreceptors is generally preserved in *Mecp2*-null mice; in particular, there are no obvious changes in the distribution of pericentromeric heterochromatin and major epigenetic markers characteristic for eu- and heterochromatin.

•MECP2 is expressed in the majority of studied 64 non-neuronal cell types; cells which do not express MECP2 are epithelial cells of the intestine, cells of the erythropoietic lineage, hair matrix keratinocytes, and mature gonads; epidermis keratinocytes express MECP2 at a very low level.

•Similarly to neurons, the expression of MECP2 in non-neuronal cells is initiated at the late differentiation stages; in this respect, gonads show a reverse pattern with no expression in differentiated oocytes and spermatozoids.

•An absence of MECP2 is not compensated by increased expression of other methyl binding proteins; in contrast, expression of some of them was downregulated.

## Methods

### Animals and primary cell cultures

All procedures were approved by the Animal Ethic Committee of Munich University and Edinburgh University. CD1, C57Bl/6, and *Mecp2*-null mice were killed by cervical dislocation according to the standard protocol. *Mecp2*^**-*/y***^ mice (described in [9]; Jackson Laboratory stock number: 003890) were generated along with wild-type littermates by crossing *Mecp2*^*+/-*^ females with wild-type male mice. The generation of mice ectopically expressing LBR in rod cells under the control of the *Nrl* promoter is described in [41]. Retinas of R7E mice [42] were studied at the age of 70 weeks. Retinas from mice with combined deletions of *Suv3-9* and *Suv4-20* were a kind gift from G. Schotta (University of Munich). Wild-type littermate controls for all genetically modified mice were studied in parallel. Tail fibroblast cell lines from *Mecp2*^-***/y***^ and *Mecp2*^***lox/y***^ mice are described in [9].

### Tissues, fixation, and cryosections

The retinas of the ICR/CD1 mice were studied on each day between E12 and P28. The retinas of *Mecp2*^**-*/y***^ mice and their WT littermates were studied at the ages of P1, P7, P14, P30, and P53. Retina fixation, embedding in freezing medium, and preparation of cryosections were performed as described previously [38,39]. Briefly, the eyes were enucleated immediately after death; the retinas were dissected and fixed with 4% formaldehyde in phosphate-buffered saline (PBS) for various times (15 min, 30 min, 1 h, 3 h, and 24 h). After washing in PBS, the samples were infiltrated in 10%, 20%, and 30% sucrose in PBS before freezing in Jung freezing medium. Importantly, the retina samples at different ages, from WT and transgenic mice, and of various fixation times, were arranged in respective order in the same block to assure identification of all retina samples in a section [39]. Retinas from monkey (*Macaca fascicularis*) and rat (*Rattus norvegicus*) were *post mortem* experimental materials from the MPI for Brain Research (Frankfurt, Germany). Other tissue samples from adult C57Bl/6 mice and rats were fixed with 4% formaldehyde in PBS for 24 h. For some tissues, the samples from different developmental stages – P0, P2, P5, P9, P14, and P28 – were used.

### Immunostaining on cryosections

Immunostaining was performed according to the protocol described in detail by [38,39]. This protocol allows quick testing of a wide range of fixation and antigen retrieval times and detection of the range in which the results of staining are robust. Antigen retrieval was crucial for robust MECP2 staining and was performed by heating cryosections in 10 mM sodium citrate buffer at 80°C. MECP2 detection after 12–24 h of tissue fixation was most successful after 20–30 min of antigen retrieval. For MECP2 immunostaining, mostly rabbit polyclonal antibodies were used. Specificity of the antibody was checked using fibroblasts derived from *Mecp2*^**-*/y***^ and *Mecp2*^***lox/y***^ mice (Additional file 1). In some cases, rat monoclonal antibodies were used as well [65]. The antibodies for cell type identification and for recognition of retinal structures are listed in Tables 1 and 3. Antibodies for the detection of histone modifications are listed in Table 2. Secondary antibodies were anti-mouse IgG conjugated to Alexa555 (A31570, Invitrogen, Renfrew, UK) or Alexa488 (A21202, Invitrogen), and anti-rabbit IgG conjugated to DyLight549 (711-505-152, Jackson ImmunoResearch, West Grove, PA, USA) or DyLight488 (711-485-152, Jackson ImmunoResearch). The nuclei were counterstained with DAPI added to the secondary antibody solution. After staining, the sections were mounted under a coverslip with Vectashield (Vector Laboratories, Inc., Burlingame, CA, USA).

### Light microscopy

Single optical sections or stacks of optical sections were collected using a Leica TCS SP5 confocal microscope (Milton Keynes, UK) equipped with Plan Apo 63×/1.4 NA oil immersion objective and lasers with excitation lines 405, 488, and 561 nm. Dedicated plug-ins in the ImageJ program were used to compensate for axial chromatic shift between fluorochromes in confocal stacks, to create RGB stacks/images, and to arrange optical sections into galleries [66,67].

### Chromocenter scoring

Chromocenters in the rod cells were scored at two age points, P30 and P53. For each age, three mice were used, two *Mecp2*^-*/y*^ and one *Mecp2*^*+/y*^ littermate. From each animal 25-μm-thick cryosections were prepared from the three retina areas: central, mid, and peripheral. To distinguish between individual nuclei in tightly packed rod perikarya, the nuclear envelope of rod cells was stained with anti-lamin B1 antibodies (sc-6217). Between 600 and 800 rod cell nuclei were scored in stacks collected from each retina area. Descriptive statistics was performed using SigmaStat software.

### RNA isolation and RT-qPCR

The tissue samples of *Mecp2*-null mice were collected in ‘RNAlater’ (Qiagen, Venlo, Netherlands) and stored at -20°C. Isolation of RNA and reverse transcription were carried out as described previously [68]. Primers for RT-qPCR were either designed with the Primer Express software (Applied Biosystems Inc., Foster City, CA, USA) or used as previously published (Table 4). RT-qPCR was performed on the 7500 Fast Real-Time PCR System (Applied Biosystems) at standard reaction conditions using the *Power* SYBR Green PCR Master Mix (Applied Biosystems). Gene expression levels were normalized to *Gapdh* and calculated using the comparative CT method (ΔΔCT method). Relative quantification of gene expression was performed by the 2^-ΔΔCT^ method based on the CT values of both target and reference genes. The results of the real-time PCR analysis of two (tissues) and three (cells) biological replicates are given as mean ± S.E.M. The statistical difference between the values was estimated by *t* test using SSPS.

**Table 4:** List of primers used for real-time PCR

**Gene**	**Forward**	**Reverse**
Mbd1*	*GAGCACAGAGAATCGCCTTC*	*CACACCCCACAGTCCTCTTT*
Mbd2*	*CTGGCAAGATACCTGGGAAA*	*TTCCGGAGTCTCTGCTTGTT*
Mbd3*	*AGAAGAACCCTGGTGTGTGG*	*TGTACCAGCTCCTCCTGCTT*
Mbd4*	*ACAGGATGGCTCTGAAATGC*	*TCTACTTGTGTCCGTGGGATG*
*Mbd5* isoform 1**	*GAGGCCATGAGCGAACTG*	*TCTTCCTCCTCTTGGGTTTG*
Mbd6**	*CCCGGGGATAGTCAGAAAGT*	*AGCTGCTCGCGTTGTAGG*
Mecp2*	*CAGGCAAAGCAGAAACATCA*	*GCAAGGTGGGGTCATCATAC*
*Zbtb33*	*ATCATTAGCTCCAGTCCAGACTCA*	*ATCTGCATCTTCTGTGTCAATGATC*
Zbtb38*	*CATCTTTTGGAGCCATACGATCT*	*TGACGGTTTCCTGTCTTTTGAC*
*Zbtb4*	*CCCTGCCGCTACTGTGAGA*	*CAGCAGAAGATGCACTGGTACCT*
*Setdb1*	*GGCCATTCCTCCCCTACTTC*	*GGCCAAAGGTGACCGATATG*
*Uhrf1*	*GGCAGCTGAAGCGGATGA*	*CCATGCACCGAAGATATTGTCA*
*Uhrf2UHRF2*	*CATGGTCGCAGCAATGATG*	*CACCGCTTCCAGTATACGTGAA*
*Gapdh*	*CATGGCCTTCCGTGTTCCTA*	*CTTCACCACCTTCTTGATGTCATC*

*Single asterisk* denotes that the sequence was taken from [64]. *Double asterisks* signify that the sequence was taken from [69].

## Abbreviations

BDNF: Brain-derived neurotrophic factor; GCL: Ganglion cell layer; INL: Inner nuclear layer; MBD: Methyl binding domain; MECP2: Methyl-CpG binding protein 2; ONL: Outer nuclear layer; OPL: Outer plexiform layer; IPL: Plexiform layer; SCA7: Spinocerebellar ataxia type 7; WT: Wild-type.

## Competing interests

The authors declare that they have no competing interests.

## Authors’ contributions

CS performed immunostainings, confocal microscopy, RT-qPCR experiments, and data analysis. YF performed immunostainings and confocal microscopy and contributed to manuscript writing. JG collected eye samples and tissues for RT-qPCR. LP supplied antibodies against neural tissues and contributed to manuscript writing. KLJ supplied anti-MECP2 antibodies and performed Western blot analysis. HK supplied antibodies against histone modifications. MCC contributed to the study design and manuscript writing. AB contributed to manuscript writing. HL contributed to manuscript writing. BJ and IS designed and supervised the study; performed immunostainings, confocal microscopy, and data analysis; and wrote the paper. All authors read and approved the final manuscript.

## Supplementary Material

Additional file 1 **Immunostaining and Western blot analysis with rabbit anti-MECP2 antibody.** Specificity of the rabbit anti-MECP2 antibody and its application for Western blot analysis of MECP2 level in different mouse tissues. Click here for file

Additional file 2 ***Mecp2***^***-/y***^**and** ***Mecp2***^***wt***^**retinas at different developmental stages.***Mecp2*^*-/y*^ and *Mecp2*^*wt*^ littermate retinas are not different with respect to the time of layer formation, thickness of nuclear and plexiform layers, and other morphological features. Click here for file

Additional file 3 **Distribution of neurons, synapses, and neurotransmitters in *Mecp2***^***wt ***^**and *Mecp2***^**-*/y ***^**retinas.** Retinas of *Mecp2*^-*/y*^ mice show no apparent defects in the distribution of neurons, synapses, and neurotransmitters in comparison to *Mecp2*^*wt*^ littermates. Click here for file

Additional file 4 **Distribution of histone modifications in ganglion and INL cells of *Mecp2***^***wt ***^**and *Mecp2***^**-*/y ***^**retinas.** Similar distribution of histone modifications characteristic of euchromatin and heterochromatin in *Mecp2*^-*/y*^ and *Mecp2*^*wt*^ mice. Click here for file

Additional file 5 **MECP2 expression in retinal cells from *Suv3-9/Suv4-20*** **double KO mice.** Similar to WT mouse retina, rods of double KO mice express MECP2 at a very low level, whereas other retinal neurons strongly express MECP2. Click here for file

Additional file 6 **Gene expression analysis of MBD proteins in *Mecp2***^***-/y***^**and wild-type mice.** Relative transcription levels of MBD proteins were determined by RT-qPCR in gut, skeletal muscles and heart of *Mecp2*^-*/y*^ and *Mecp2*^*wt*^ mice. Click here for file
