# Supplementary material for: DNA methylation reader MECP2: cell type- and differentiation stage-specific protein distribution
Source: Epigenetics Chromatin. 2014 Aug 3;7:17. doi: 10.1186/1756-8935-7-17 (PMC4148084; doi:10.1186/1756-8935-7-17)
Supplement: Additional file 1 — Immunostaining and Western blot analysis with rabbit anti-MECP2 antibody. Specificity of the rabbit anti-MECP2 antibody and its application for Western blot analysis of MECP2 level in different mouse tissues. [file 1756-8935-7-17-S1.pdf]

Additional file 1.

Specificity of rabbit-anti-MECP2 antibody (A) and its application for Western blot analysis of MECP2 level in different mouse tissues (B).

A. Immunostaining of cultured primary fibroblasts derived from *Mecp2*$^{-/y}$ and *Mecp2*$^{lox/y}$ mice. Chromocenters are stained with rabbit-anti-MECP2 antibody (green) in *Mecp2*$^{lox/y}$ but not in *Mecp2*$^{-/y}$ fibroblast nuclei which indicates to specificity of the antibody used. Chromocenters (arrows) comprise AT-rich major satellite repeat and consequently brightly stained with DAPI (red).

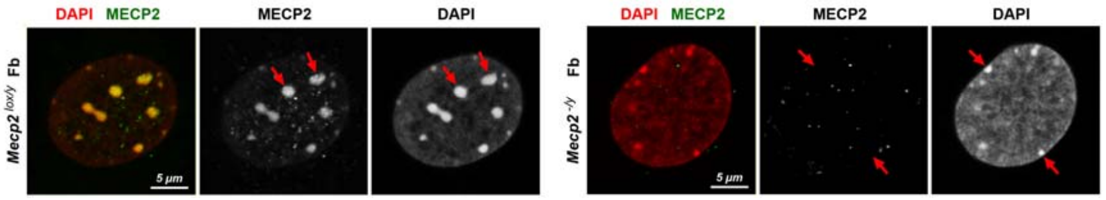

B. Western blot analysis of MECP2 level in different mouse tissues.

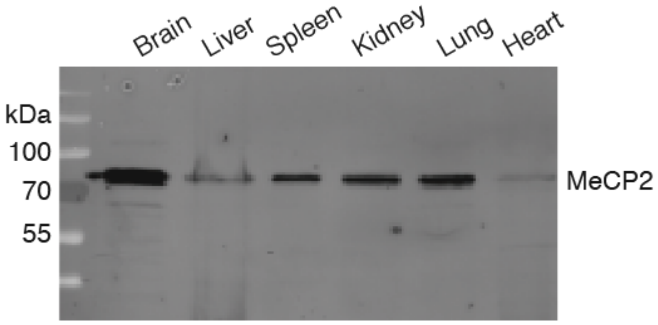

Nuclei were isolated as previously described (*Prusov, A.N. and Zatsepina, O.V. (2002) Isolation of the chromocenter fraction from mouse liver nuclei. Biochemistry (Mosc), 67, 423-431*). In short, mouse tissues were dissected, frozen with liquid nitrogen and pulverized using a mortar (3g tissue each), and homogenized in 15 ml buffer A (20 mM TEA, 30 mM KCl, 10 mM $MgCl_2$, 0.25 M sucrose) using a douncer. The homogenate was centrifuged for 10 min at 1000g using a Eppendorf Centrifuge 5810 R (rotor A 462), The resulting pellets were resuspended in buffer B (identical with A but containing 2.5 M sucrose) to a final concentration of 2.1 M sucrose, and centrifuged at 50.000g (SW28 rotor in a Beckmann ultracentrifuge L8-70 M") for 40 min with slow deceleration/acceleration. Resulting nuclei pellets were washed in 20 ml of buffer A and centrifuged at 1000 g for 10 min. All steps were carried out at 4°C. The obtained pellet contained mostly nuclei; the purity of the fraction was controlled by phase contrast microscopy (Zeiss Axioplan 200). Nuclei isolation yielded approx. $1x10^7$ (brain, spleen, lung) and $1x10^8$ nuclei (liver, kidney, heart) per sample. Proteins were extracted using RIPA buffer and subsequently boiled in Laemmli sample buffer. Proteins were separated on a 10% SDS-PAGE and western blot analysis was performed using affinity purified rabbit polyclonal anti-MeCP2 antibody (1:500) and secondary anti-rabbit antibody conjugated to Alexa 488 (*Jost KL, Rottach A, Milden M, Bertulat B, Becker A, et al. (2011) Generation and Characterization of Rat and Mouse Monoclonal Antibodies Specific forMeCP2 and Their Use in X-Inactivation Studies. PLoS ONE 6(11): e26499*).
