# Supplementary material for: DNA methylation reader MECP2: cell type- and differentiation stage-specific protein distribution
Source: Epigenetics Chromatin. 2014 Aug 3;7:17. doi: 10.1186/1756-8935-7-17 (PMC4148084; doi:10.1186/1756-8935-7-17)

<u>Additional file 2</u>.

***Mecp2*$^{-/y}$ and *Mecp2*^{wt} littermate retinas are not different with respect to the time of the layers formation, thickness of nuclear and plexiform layers, and other morphological features at postnatal ages P1, P7, P13, P30, and P53.**

ONL, outer nuclear layer; INL, inner nuclear layer; GCL, ganglion cell layer; IPL, inner plexiform layer; OPL, outer plexiform layer. Single confocal sections. Scale bar: 50 µm

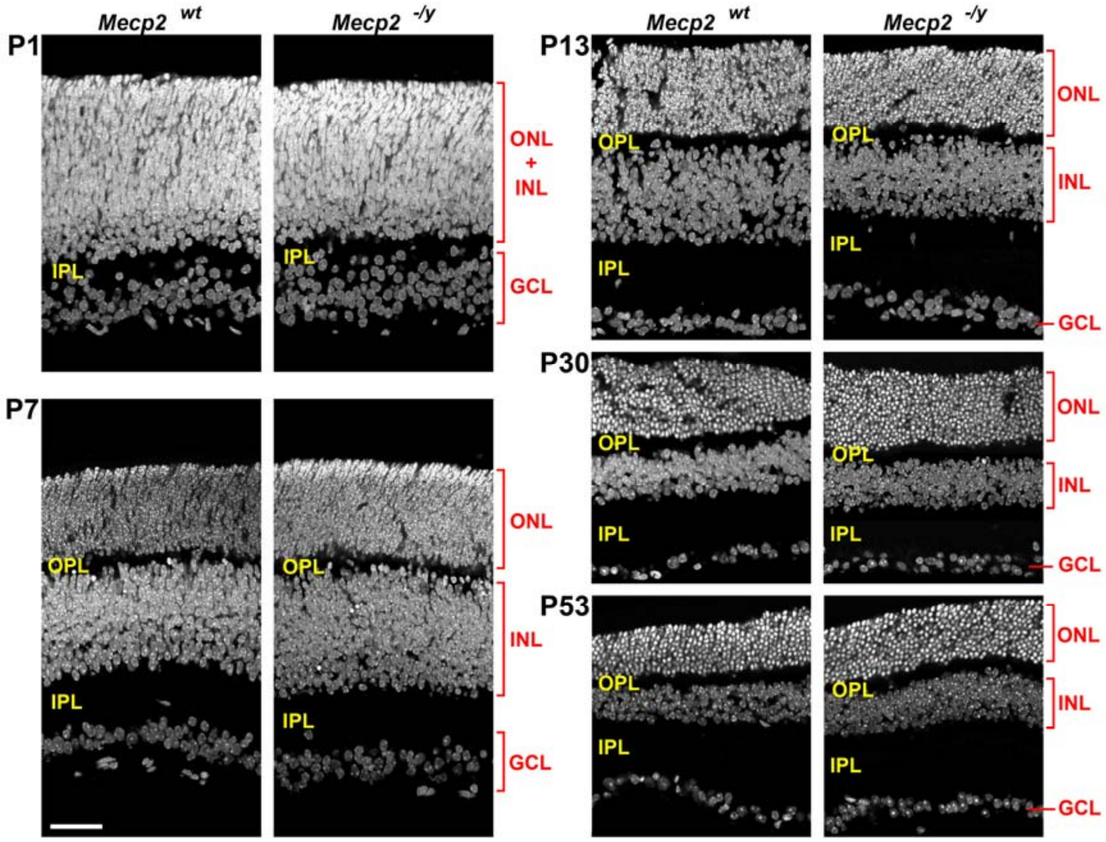

Supplement: Additional file 2 — Mecp2 -/y and Mecp2 wt retinas at different developmental stages.Mecp2 -/y and Mecp2 wt littermate retinas are not different with respect to the time of layer formation, thickness of nuclear and plexiform layers, and other morphological features. [file 1756-8935-7-17-S2.pdf]
