# Supplementary material for: DNA methylation reader MECP2: cell type- and differentiation stage-specific protein distribution
Source: Epigenetics Chromatin. 2014 Aug 3;7:17. doi: 10.1186/1756-8935-7-17 (PMC4148084; doi:10.1186/1756-8935-7-17)

Additional file 3 (A, B).

Retinas of *Mecp2*$^{-/y}$ mice show no apparent defects in the distribution of neurons, synapses, and neurotransmitters in comparison to *Mecp2*^{wt} littermates

Four other marker stainings are shown on Figure 3A. See Table 1 for structures selectively marked by immunostaining and for the source of the antibodies.

Additional file 3A:

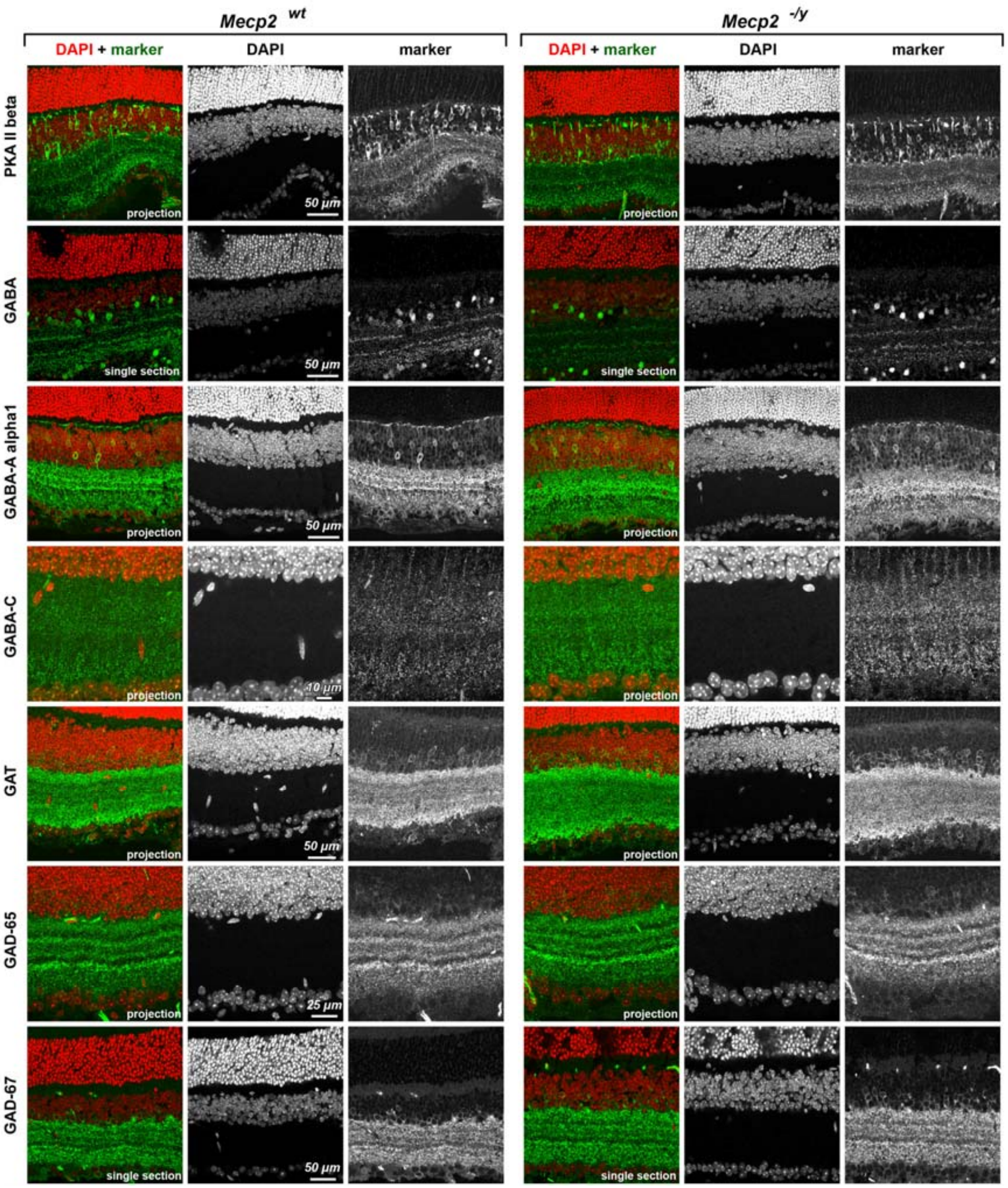

Additional file 3B:

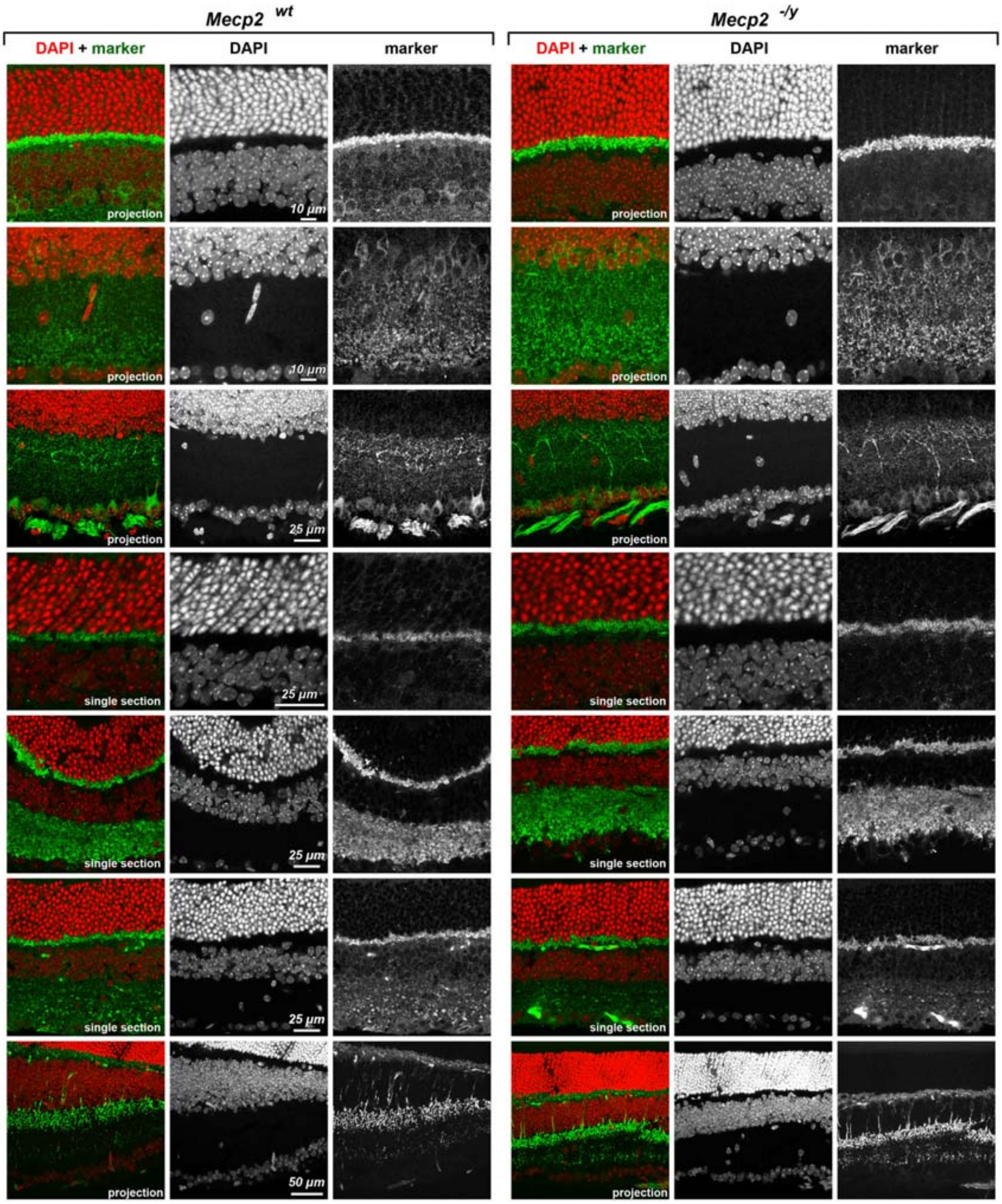

Supplement: Additional file 3 — Distribution of neurons, synapses, and neurotransmitters in Mecp2 wt and Mecp2 - /y retinas. Retinas of Mecp2-/y mice show no apparent defects in the distribution of neurons, synapses, and neurotransmitters in comparison to Mecp2 wt littermates. [file 1756-8935-7-17-S3.pdf]
