# Supplementary material for: DNA methylation reader MECP2: cell type- and differentiation stage-specific protein distribution
Source: Epigenetics Chromatin. 2014 Aug 3;7:17. doi: 10.1186/1756-8935-7-17 (PMC4148084; doi:10.1186/1756-8935-7-17)

Additional file 4.

Similar distribution of histone modifications characteristic of euchromatin and heterochromatin in *Mecp2* $^{-/y}$ and *Mecp2* ^{wt}

Nuclei with conventional architecture, ganglion and INL cells, are shown. Histone modifications and DAPI nuclear counterstain are shown in green and red, respectively. Single confocal sections. Scale bars: 5 µm

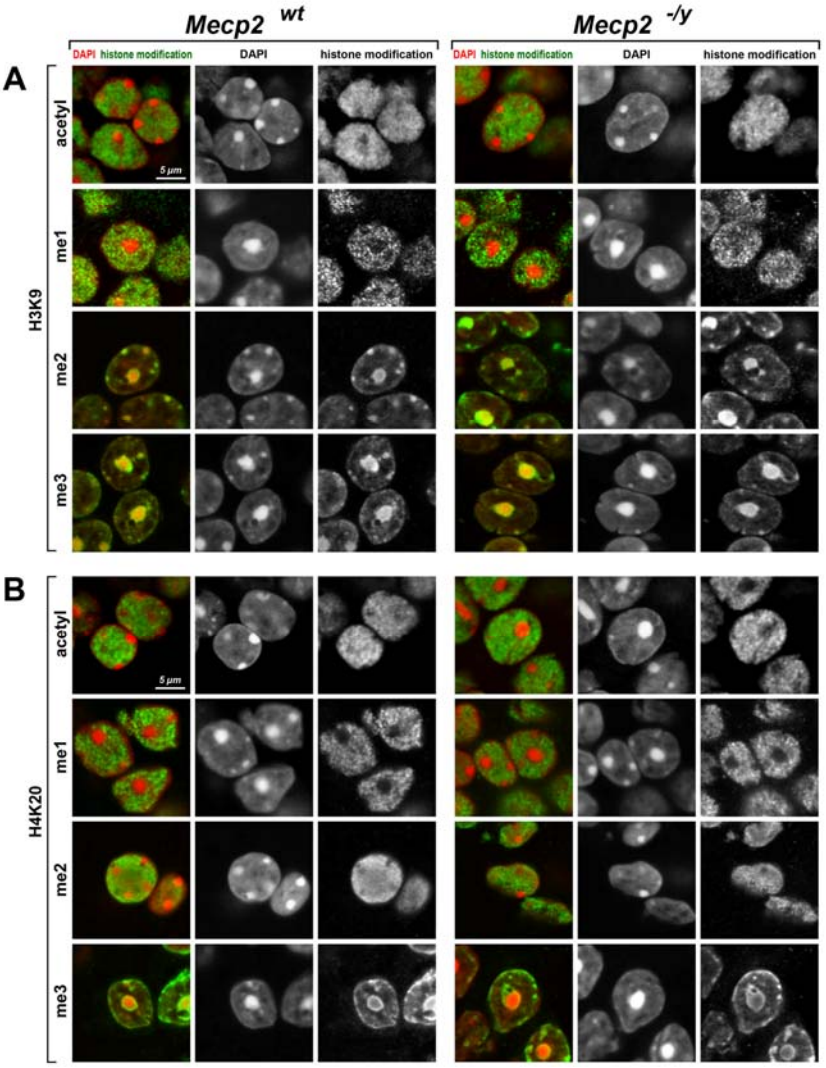

Supplement: Additional file 4 — Distribution of histone modifications in ganglion and INL cells of Mecp2 wt and Mecp2 - /y retinas. Similar distribution of histone modifications characteristic of euchromatin and heterochromatin in Mecp2-/y and Mecp2 wt mice. [file 1756-8935-7-17-S4.pdf]
