# Supplementary material for: DNA methylation reader MECP2: cell type- and differentiation stage-specific protein distribution
Source: Epigenetics Chromatin. 2014 Aug 3;7:17. doi: 10.1186/1756-8935-7-17 (PMC4148084; doi:10.1186/1756-8935-7-17)

<u>Additional file 5</u>.

Expression of MECP2 in retinal cells from double KO mice with combined deletion of *Suv3-9* and *Suv4-20*

Similar to WT mouse retina, rods of double KO mice (arrowheads) express MeCP2 at a very low level, whereas cones (arrows) in ONL and other neurons from INL and GCL strongly express MeCP2. Note typical distribution of MeCP2 in nuclei (compare with WT retina cells on Figure 1A). Single confocal sections. Scale bars: upper panel, 25 µm; middle and lower panels, 5 µm

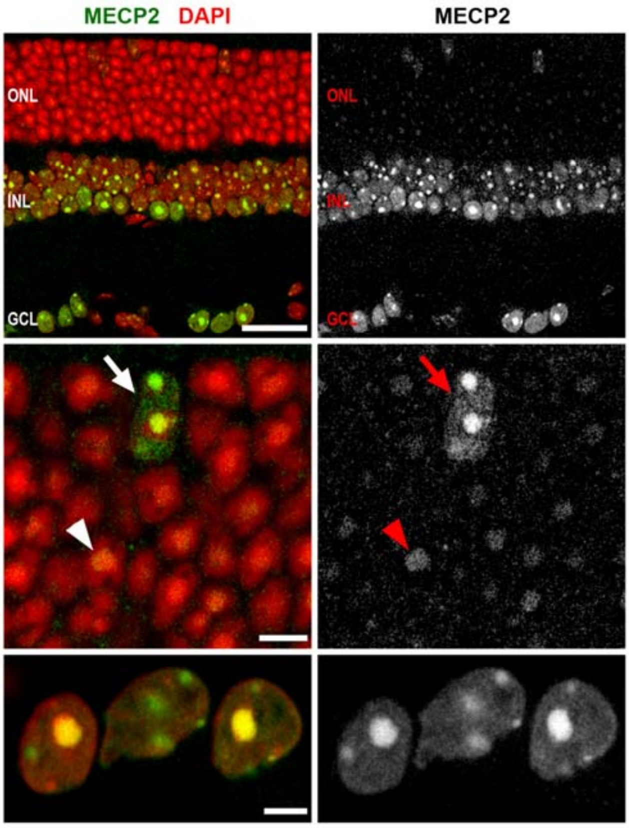

Supplement: Additional file 5 — MECP2 expression in retinal cells from Suv3-9/ Suv4-20 double KO mice. Similar to WT mouse retina, rods of double KO mice express MECP2 at a very low level, whereas other retinal neurons strongly express MECP2. [file 1756-8935-7-17-S5.pdf]
