# Supplementary material for: DNA methylation reader MECP2: cell type- and differentiation stage-specific protein distribution
Source: Epigenetics Chromatin. 2014 Aug 3;7:17. doi: 10.1186/1756-8935-7-17 (PMC4148084; doi:10.1186/1756-8935-7-17)

Additional file 6.

Analysis of expression of MBD proteins in three tissues from *Mecp2*$^{-/y}$ and *Mecp2*^{wt} mice

Relative transcription level of MBD proteins in gut (A), skeletal muscles (B), and heart (C) from *Mecp2*$^{-/y}$ and littermate *Mecp2*^{wt} mice. Values are normalized to transcript of *Mecp2* in *Mecp2*^{wt} of respective tissue. Note that there are no statistically significant changes in transcription of MBD protein genes upon deletion of *Mecp2*.

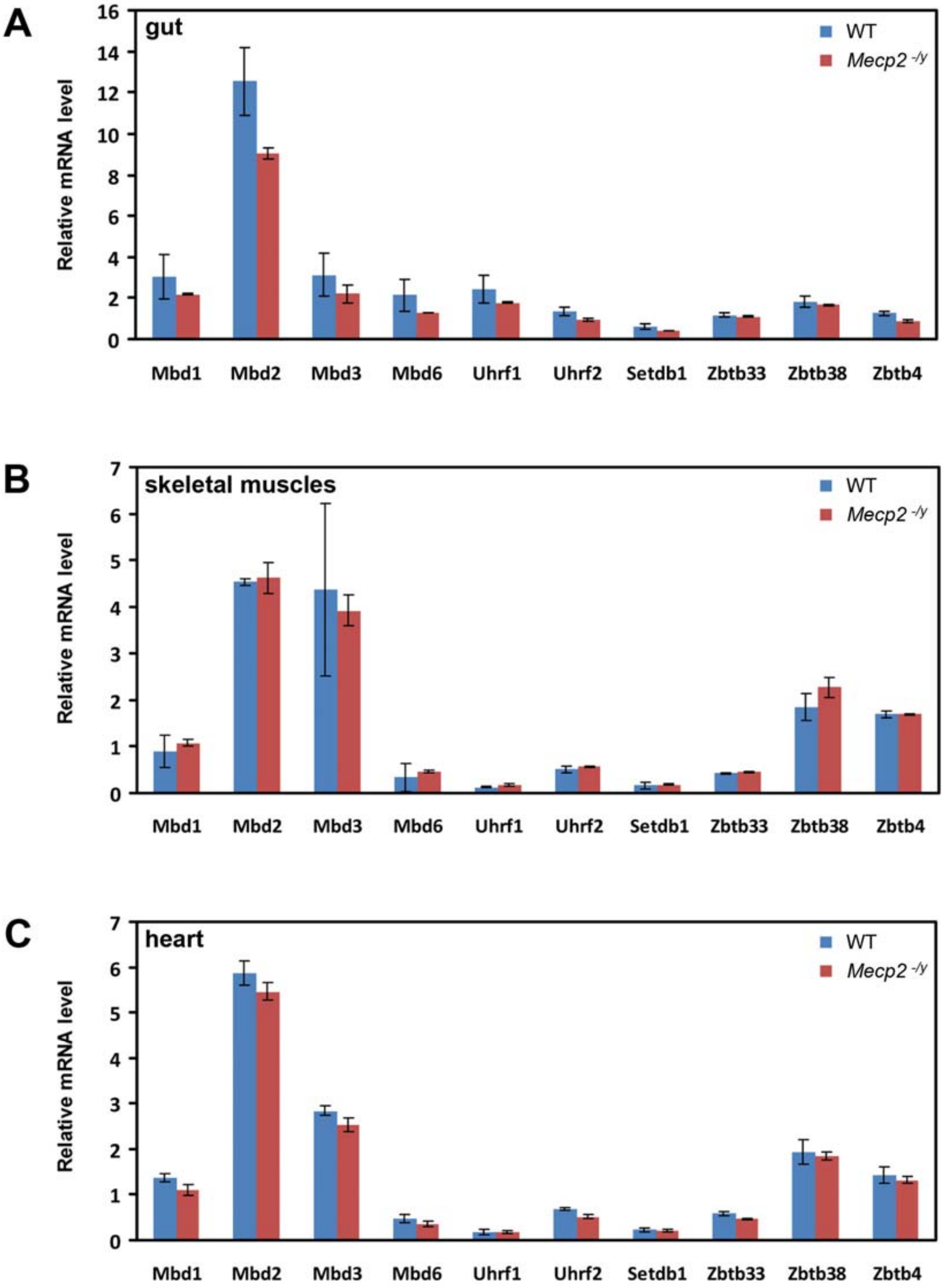

Supplement: Additional file 6 — Gene expression analysis of MBD proteins in Mecp2 - /y and wild-type mice. Relative transcription levels of MBD proteins were determined by RT-qPCR in gut, skeletal muscles and heart of Mecp2-/y and Mecp2 wt mice. [file 1756-8935-7-17-S6.pdf]
